# Targeted Delivery to Dying Cells Through P-Selectin–PSGL-1 Axis: A Promising Strategy for Enhanced Drug Efficacy in Liver Injury Models

**DOI:** 10.3390/cells13211778

**Published:** 2024-10-27

**Authors:** Te-Sheng Lien, Der-Shan Sun, Hsin-Hou Chang

**Affiliations:** Department of Molecular Biology and Human Genetics, Tzu-Chi University, Hualien 970, Taiwan; alan211@mail.tcu.edu.tw (T.-S.L.); dssun@mail.tcu.edu.tw (D.-S.S.)

**Keywords:** dying-cell-specific drug delivery system, drug-induced liver damage, fluorescent dye delivery, image tracking and diagnosis, apoptosis inhibitor z-DEVD, regulated cell death

## Abstract

To minimize off-target adverse effects and improve drug efficacy, various tissue-specific drug delivery systems have been developed. However, even in diseased organs, both normal and stressed, dying cells coexist, and a targeted delivery system specifically for dying cells has yet to be explored to mitigate off-target effects within the same organ. This study aimed to establish such a system. By examining the surfaces of dying cells in vitro, we identified P-selectin glycoprotein ligand-1 (PSGL-1) as a universal marker for dying cells, positioning it as a potential target for selective drug delivery. We demonstrated that liposomes conjugated with the PSGL-1 binding protein P-selectin had significantly greater binding efficiency to dying cells compared to control proteins such as E-selectin, L-selectin, galectin-1, and C-type lectin-like receptor 2. Using thioacetamide (TAA) to induce hepatitis and hepatocyte damage in mice, we assessed the effectiveness of our P-selectin-based delivery system. In vivo, P-selectin-conjugated liposomes effectively delivered fluorescent dye and the apoptosis inhibitor z-DEVD to TAA-damaged livers in wild-type mice, but not in PSGL-1 knockout mice. In TAA-treated wild-type mice, unconjugated liposomes required a 100-fold higher z-DEVD dose compared to P-selectin-conjugated liposomes to achieve a comparable, albeit less effective, therapeutic outcome in lowering plasma alanine transaminase levels and alleviating thrombocytopenia. This emphasizes that P-selectin conjugation enhances drug delivery efficiency by approximately 100-fold in mice. These results suggest that P-selectin-based liposomes could be a promising strategy for targeted drug delivery, enabling both diagnosis and treatment by specifically delivering cell-labeling agents and rescue agents to dying cells via the P-selectin–PSGL-1 axis at the individual cell level.

## 1. Introduction

Tissue-specific targeted drug delivery systems are well-established and have been extensively studied in various models [1,2,3,4,5,6,7,8,9,10,11,12,13,14,15,16,17,18,19]. However, a drug delivery system that specifically targets dying cells could present unique advantages. Firstly, it may improve drug specificity by directing the therapeutic agents exclusively to dying cells, thereby reducing off-target effects and enhancing overall treatment efficacy. Secondly, this approach could lower drug toxicity by decreasing the required dosage, which would also minimize potential side effects. Thirdly, such a system has the potential to improve treatment outcomes across a broad spectrum of diseases associated with cell death, including tissue damage and degeneration. Additionally, it aligns with personalized medicine by customizing drug delivery according to an individual’s specific cell-death profile. Despite its potential, a dying-cell-targeting drug delivery system is yet clinically available, and warrants further investigation.

Apoptosis, a type of regulated cell death (RCD), is crucial for maintaining tissue homeostasis and is typically non-inflammatory and essential for health [20,21]. However, excessive apoptosis has been implicated in various diseases [22]. Consequently, a drug delivery system targeting apoptotic cells could specifically enhance therapeutic effectiveness in affected tissues while reducing unintended effects on normal cells. In this study, we discovered that different RCD inducers can lead to the expression of P-selectin-ligand-1 (PSGL-1) on the surface of dying cells. This finding suggests that counter receptors capable of binding to PSGL-1, such as selectins, could be used to develop a dying-cell-targeting drug delivery system. Supporting this observation, in vitro experiments showed that selectin-conjugated, fluorescein-loaded liposomes were more readily engulfed by dying cells than by normal cells, which did not uptake the liposomes at all. This provides initial evidence that selectin-conjugated liposomes could be a promising tool for delivering drugs to dying cells.

To assess the in vivo efficacy of this drug delivery system, we employed a thioacetamide (TAA)-induced hepatitis mouse model, which is widely used to study drug-induced hepatitis because it replicates key aspects of human liver disease, such as acute liver injury, inflammation, oxidative stress, and apoptosis of liver cells [23,24,25,26,27]. This model in part resembles the pathology of human liver diseases associated with apoptosis, such as alcoholic liver disease and drug-induced liver injury, where apoptosis leads to hepatocyte destruction and liver dysfunction [28,29,30]. To investigate hepatocyte-specific responses, we used asialoglycoprotein receptor 1 (ASGR1) as a hepatocyte surface marker [31,32,33]. A fluorescence-labeled antibody targeting ASGR1, the primary subunit of ASGPR (also known as the Ashwell–Morell receptor or hepatic lectin), enabled us to study liver cell behavior via flow cytometry under TAA challenge and evaluate the reversal effects of z-DEVD apoptosis inhibitor delivery through our P-selectin-based dying-cell-targeting system in mice.

Our study demonstrated that this selectin-conjugated, liposome-based drug delivery system effectively mitigated TAA-induced hepatitis in mice, highlighting its therapeutic potential. The underlying mechanisms and potential applications of this system are discussed.

## 2. Materials and Methods

### 2.1. Experimental Mice

Wild-type C57BL/6J mice (8 to 12 weeks old) were obtained from the National Laboratory Animal Center in Taipei, Taiwan. PSGL-1 knockout (KO; *Selplg*^−/−^; B6.129-Selplg tm1Rpmc/J) mice were sourced from the Jackson Laboratory (Bar Harbor, ME, USA) [34]. These *Selplg*^−/−^ mice were backcrossed with wild-type C57BL/6J mice for six generations. The genotype of the PSGL-1 KO mice was routinely verified every 15 to 20 generations, following the Jackson Laboratory protocol (https://www.jax.org/Protocol?stockNumber=006336&protocolID=22551, accessed 20 September 2024). All mice were housed in the Tzu-Chi University Animal Center in a specific pathogen-free environment with controlled light and temperature and provided with food and filtered water. A total of 287 wild-type mice and 131 *Selplg*^−/−^ mice were used. The experimental protocols were approved by the Animal Care and Use Committee of Tzu-Chi University, Hualien, Taiwan (approval ID: 112031).

Primary mouse splenocytes were isolated as described in [35,36] by cutting spleen samples into small pieces and incubating them with 1 mg/mL collagenase D (Sigma-Aldrich, Burlington, MA, USA) in serum-free cell culture medium for 30 min at 37 °C with shaking in a 15 mL falcon tube. Dissociation of splenocytes from cell and tissue pellets was achieved using 2 mL of non-enzymatic cell dissociation solution (Sigma-Aldrich) at room temperature for 10 min [36]. After washing with PBS to remove the cell dissociation solution, the splenocytes were resuspended in PBS and immediately used for in vitro experiments without being plated on culture dishes.

The isolation of liver cells proved more challenging than splenocytes for flow cytometry. To prepare mouse liver cell suspensions, the liver was perfused with PBS through the vena cava, following previously described methods [37,38]. The liver was then dissociated and incubated in 2 mL of non-enzymatic cell dissociation solution (Sigma-Aldrich) with dispase II (1 U/mL, Sigma-Aldrich) at 37 °C for 10 min. After filtration through a 100 nm mesh to remove large tissue debris, the liver cell suspension was subjected to Percoll-based (45%) density separation and red blood cell lysis [37,38], making the cells ready for subsequent flow cytometry analysis.

### 2.2. Mouse Model of TAA-Induced Liver Damage

The TAA-induced liver injury mouse model was established following previously described protocols [26]. Mice in the experimental groups received a single intraperitoneal injection of TAA at a dose of 45 mg/kg (dissolved in 1× sterile saline solution at 100 mg/mL) to induce acute hepatitis. Modified from reported experiments [39,40,41], z-DEVD-loaded liposomes (5 × 10^7^ liposomes, containing 2 μM z-DEVD/mouse) were administered intravenously 24 h after TAA treatment. After 48 h of TAA exposure, whole blood, plasma, and liver samples were collected for analysis. Mice in the control group received injections of sterile saline solution. To obtain blood and plasma samples, mouse blood was collected and transferred into polypropylene tubes containing an anticoagulant solution of acid–citrate–dextrose (38 mM citric acid, 75 mM sodium citrate, and 100 mM dextrose) [34]. The collected samples were analyzed for platelet count, alanine transaminase (ALT) levels, flow cytometry, and liver imaging. Platelet counts were measured using a hematology analyzer (KX-21, Sysmex, Singapore), ALT levels were assessed with a clinical biochemistry system (COBAS INTEGRA 800, Roche Taiwan, Taipei, Taiwan), flow cytometry was performed using a flow cytometer (Gallios, Beckman Coulter Life Sciences, Brea, CA, USA), and liver imaging was conducted with the Thermo iBright FL1500 Imaging System (Thermo Fisher Scientific, Waltham, MA, USA). The TAA used in the experiments was obtained from Sigma-Aldrich (163678, St. Louis, MO, USA).

### 2.3. Liposome Preparation, Surface Protein Conjugation, Cargo Loading, and Characterizations

We prepared liposomes using a liposome kit (L4395, Sigma-Aldrich), a lyophilized powder composed of egg yolk phosphatidylcholine, stearylamine, and cholesterol, following the manufacturer’s instructions. After agitating the liposomes for 30 min, we incorporated 3-(N-succinimidyloxyglutaryl) aminopropyl, polyethyleneglycol-carbamyl distearoylphosphatidyl-ethanolamine (DSPE-PEG-NHS, MW 2000, BP-26221, BroadPharm, San Diego, CA, USA) into the lipid mixture for protein conjugation. The phosphatidylcholine, stearylamine, cholesterol, and DSPE-PEG-NHS were combined in a 14:4:2:1 ratio and heated at 60 °C for one hour. The DSPE-PEG-NHS liposomes were subsequently incubated with various proteins, including immunoglobulin G (IgG)-Fc (366902, Biolegend, San Diego, CA, USA), P-selectin (737-PS, R&D Systems, Minneapolis, MN, USA), E-selectin (575-ES, R&D Systems), L-selectin (576-LS, R&D Systems), galectin-1 (1245-CF, R&D Systems), C-type lectin-like receptor 2 (CLEC2) (1718-CL, R&D Systems), and anti-PSGL-1 antibody (7404-PS, BD Biosciences, East Rutherford, NJ, USA) at a concentration of 2 ng per 10^6^ liposomes at 37 °C for 3 h for surface protein conjugation. P-selectin, E-selectin, L-selectin, galectin-1, and CLEC2 are all IgG-Fc fusion recombinant proteins containing an IgG-Fc region, thus an isotype-matched IgG-Fc was used as the control protein. To assess protein conjugation efficiency, we collected both the supernatant and liposome fractions following the 3 h protein conjugation process. Protein concentrations were then measured using a Nanodrop spectrophotometer (2000/2000c, Thermo Scientific, Wilmington, DE, USA). First, a standard protein solution at 100 μM was prepared and serially diluted to concentrations of 50 μM, 25 μM, 12.5 μM, 6.25 μM, and 3.125 μM. The absorbance of each diluted sample was measured at 280 nm using the NanoDrop. These absorbance values were plotted against their respective protein concentrations to generate a standard curve. Using this standard curve, the amount of unbound protein in the supernatant was determined. Accordingly, protein conjugation efficiency to liposomes was determined using the formula Conjugation efficiency % = (Total − Free)/Total, with IgG-Fc and P-selectin showing an efficiency of approximately 70–75% (Figure S1).

To conduct in vitro cell–liposome engagement experiments, liposomes containing NBD-PE [N-(7-Nitrobenz-2-Oxa-1,3-Diazol-4-yl)-1,2-Dihexadecanoyl-sn-Glycero-3-Phosphoethanolamine, Triethylammonium Salt, N360, Invitrogen] were used. These NBD-PE-incorporated liposomes were prepared following a modified protocol. After agitating the liposomes (phosphatidylcholine, stearylamine, cholesterol, and NBD-PE) for 30 min, DSPE-PEG-NHS was added to the lipid mixture for protein conjugation. The components (phosphatidylcholine, stearylamine, cholesterol, NBD-PE, DSPE-PEG-NHS) were mixed in a 3150:900:450:1:325 ratio and heated at 60 °C for one hour. Subsequent protein conjugation (e.g., P-selectin) followed the described protocol in the previous sections.

According to described methods [34], liposome quantification was performed by mixing a known amount of 6 µm reference beads (polystyrene latex beads, Sigma-Aldrich) with an unknown number of liposomes, and determining the bead-to-liposome ratio using flow cytometry (Figure S2). To analyze the particle size of liposomes, 10 μL of liposome solution (5 × 10^6^ particles/µL) was added to 790 μL of 1× PBS to achieve a 1/80 dilution. It is important to note that a high dilution ratio (>1/400) may cause calibration issues and sometimes result in undetectable measurements, while a low dilution ratio (<1/16) can significantly increase aggregation (particles > 10 μM), leading to misinterpretation. The diluted sample was placed in a cuvette, and the particle size was analyzed using a particle size analyzer (ELSZ-2000, Otsuka Electronics, Osaka, Japan) as shown in Figure S3. The encapsulation efficiency (EE%) of various liposome formulations, including total fluorescein, fluorescein-loaded liposomes [Lipo(F)], fluorescein-loaded PEG-liposomes [PEG-lipo(F)], fluorescein-loaded IgG-Fc-conjugated PEG-liposomes [IgG-Fc-PEG-lipo(F)], and fluorescein-loaded P-selectin-conjugated PEG-liposomes [P-sel-PEG-lipo(F)], was calculated using the formula EE% = [(total drug added − free non-entrapped drug)/total drug added] (Figure S4).

Materials like fluoromethyl ketone (FMK)-derivatized peptides z-DEVD-FMK (200 μM, FMK004, R&D Systems, Minneapolis, MN, USA), DEVD-FMK conjugated to sulforhodamine (Red-DEVD-FMK, 100 μM, ab65617, Abcam, Cambridge, UK), Alexa Fluor 647 (1.6 mM, A33084, Invitrogen, Carlsbad, CA, USA), and fluorescein (1.5 mM, 46955, Sigma-Aldrich) were incorporated during the 30 min agitation phase at molecular ratios of 14:4:2:0.03. After ultracentrifugation (30,000× *g*, 2 h, 4 °C; Optima MAX-XP, Beckman Coulter, Brea, CA, USA) and washing to remove any unencapsulated drugs, the amount of drug encapsulated in the liposomes was determined by measuring the fluorescence intensity of the loaded materials, compared against standard curves for z-Red-DEVD-FMK, Alexa Fluor 647, and fluorescein.

### 2.4. Cell Culture and In Vitro Analyses

The primary mouse cells cannot be maintained in long-term in vitro culture, leading to continuous cell death, which results in a high background of cell death when analyzing relevant parameters. Therefore, we used a human hepatoma cell line Huh-7 as an alternative for preliminary testing. In addition, to minimize additional noise from cell death signals, we streamlined the experiment by omitting extra plating protocols. Human hepatoma cell line Huh-7, mouse melanoma cell line B16F10, and mouse macrophage cell line J774A.1 were cultured in Dulbecco’s modified Eagle’s medium containing 10% fetal bovine serum, following previously established protocols [34,42,43]. The different cell lines were exposed to the RCD inducers for a duration of 4 h. Approximately 1 × 10⁵ cells were seeded in each well of a 6-well culture dish. Following an additional 15 h of incubation, the cells were then subjected to the RCD inducer treatments. To assess PSGL-1 expression in cells treated with an RCD inducer, we incubated 1 × 10⁵ cells with PerCP-Cy™5.5 rat anti-mouse CD162 (final concentration: 1 μg/mL, 564310, Thermo Fisher Scientific) at 37 °C for 1 h. After incubation, we centrifuged the cells at 1000× *g* for 5 min and then washed them with 1× PBS. The wash and centrifugation steps were repeated twice more. Finally, the cells were resuspended in 1× PBS and analyzed using flow cytometry (Gallios, Beckman Coulter Life Sciences). To assess RCD responses induced by various stimuli, cells were incubated with different RCD inducers for 4 h: staurosporine (10 μM [44,45]; apoptosis, 81590, Cayman Chemical, Ann Arbor, MI, USA), cisplatin (50 μM [46,47]; apoptosis, 13119, Cayman Chemical), 5-Fu (50 μM [48]; apoptosis, 14416, Cayman Chemical), rapamycin (2 μM [49,50]; autophagy, 13346, Cayman Chemical), erastin (20 μM [51]; ferroptosis, 17754, Cayman Chemical), TNF-α (0.2 μM [52,53]; necroptosis, 410-MT, R&D Systems), and nigericin (5–10 μM [54,55]; pyroptosis, 11437, Cayman Chemical). The F4/80^+^ population in splenocytes was detected using PE anti-mouse F4/80 antibody (BioLegend, San Diego, CA) and analyzed with a flow cytometer (Gallios, Beckman Coulter Life Sciences) to determine surface PSGL-1 expression.

For the liposome–cell engagement analysis, we employed two distinct systems. For Huh-7 cells, we utilized NBD-incorporated liposomes to confirm that the liposomes were actually internalized by the cells, rather than merely binding to the surface. First, Huh-7 cells were treated with apoptosis inducers at 37 °C for 3 h. These apoptotic Huh-7 cells (1 × 10^5^/mL) were then incubated with various protein-conjugated NBD–liposomes (1 × 10^5^/mL) at 37 °C for 1 h. To distinguish internalized liposomes, we added the quenching reagent dithionite (5 μM; 157953, Sigma-Aldrich), which selectively quenches NBD fluorescence on the cell surface while sparing internalized NBD. After incubating at 25 °C for 90 s and washing with PBS, samples were analyzed by flow cytometry. Examples of flow cytometry gating of NBD–liposome experiments were indicated (Figure S5).

For primary mouse liver cells, we incubated the cells (1 × 10^5^/mL) with Alexa Fluor 647-loaded liposomes (1 × 10^5^/mL) at 37 °C for 1 h in DMEM. Following washing to remove unbound liposomes, flow cytometry was performed using a fluorescence-labeled anti-ASGR1 antibody (Thermo Fisher Scientific) to quantify the ASGR1^+^Alexa Fluor 647^+^ double-positive population, indicating successful cell–liposome interaction. Examples of flow cytometry gating of Alexa Fluor 647-loaded liposome–cell engagement experiments were indicated (Figure S6).

### 2.5. RCD Profiling, Cell Death Inhibitor, and Analyses

The apoptosis inhibitor z-DEVD (10 μM, R&D Systems, Indianapolis, IN, USA) was used to address the involvement of apoptosis and its application in the reversal of TAA-induced liver damage. To detect TAA-induced RCD profile in the liver cells using flow cytometry, various RCD responses were analyzed using specific labeling reagents, including apoptosis (1 h, Red-DEVD-FMK, ab65617, Abcam), autophagy (1 h, Cyto-ID™ Autophagy Detection Kit, ENZ-51031, Enzo Life Sciences, NY, USA), ferroptosis (1 h, C11 BODIPY 581/591, 27086, Cayman Chemical), necroptosis (1 h, RIP3/B-2 Alexa Fluor 488, sc-374639 AF488, Santa Cruz Biotechnology, CA, USA), pyroptosis (1 h, Caspase-1 Assay, Green-Fluorochrome-Labeled Inhibitors of Caspases [FLICA], 9146, ImmunoChemistry Technologies, MI, USA; Caspase-1 Colorimetric Assay Kit, BioVision, CA, USA), and live/dead cell labeling (Zombie NIR™ Fixable Viability Kit, 423105, Biolegend). These were performed using the respective reagents for 30 min in PBS, following established protocols [56,57,58,59].

### 2.6. Imaging Analysis of Fluorescent Dye Delivered to Liver

To assess targeted delivery to the TAA-damaged liver, mice were injected with TAA at a dose of 45 mg/kg (dissolved in 1× sterile saline solution at 100 mg/mL) to induce acute hepatitis. Forty-five hours later, fluorescein-loaded P-selectin-conjugated liposomes (Sigma-Aldrich) were intravenously administered at a dosage of 5 × 10^7^ liposomes per mouse. Liver samples were collected 48 h post-TAA treatment from sacrificed mice, and liver imaging was performed using the Thermo iBright FL1500 Imaging System (Thermo Fisher Scientific, Waltham, MA, USA). The relative fluorescence intensities of the liver images were quantified using ImageJ software (version 1.32; National Institutes of Health, Bethesda, MD, USA) [59].

### 2.7. Statistical Analyses

The experimental data were analyzed using Microsoft Office Excel 2003 and SPSS 17 and are presented as mean ± standard error. Statistical significance was evaluated through a one-way analysis of variance (ANOVA) followed by a post-hoc Bonferroni-corrected *t*-test. A significance level of α = 0.05 was set to determine statistical significance while accounting for the probability of type 1 error.

## 3. Results

### 3.1. PSGL-1 Is Commonly Expressed on the Surfaces of Stressed, Dying Cells

**Figure 1:**
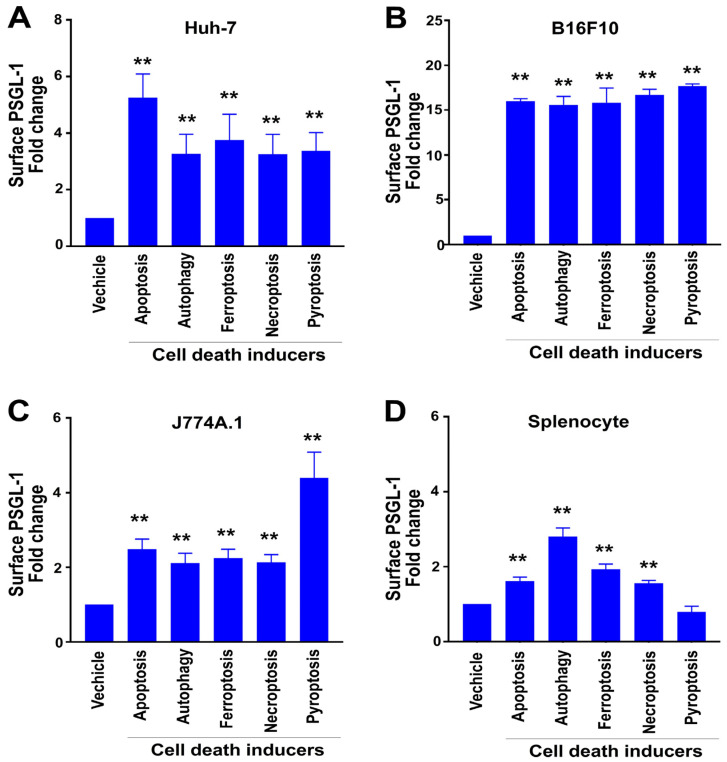
Measurement of relative surface PSGL-1 expression after F4/80^+^ splenocytes, Huh-7, B16F10, and J774A.1 cells were treated with cell death inducers. (**A**) Human hepatoma cell line Huh-7, (**B**) mouse melanoma cell line B16F10, (**C**) mouse macrophage cell line J774A.1, and (**D**) primary F4/80^+^ splenocytes were treated with inducers of apoptosis (staurosporine, 10 μM), autophagy (rapamycin, 2 μM), ferroptosis (erastin, 20 μM), necroptosis (TNF-α, 0.2 μM), and pyroptosis (nigericin, 10 μM) for 4 h. Surface PSGL-1 expression levels were then measured by flow cytometry. Vehicle control groups were normalized to 1-fold. ** *p* < 0.01 indicates markedly increased vs. respective vehicle-treated groups. The study included three experiments with two replicates per group [*N* = 3 (3 independent experiments), with *n* = 2 (2 technical replicates per experiment)].

We treated various cell types, including Huh-7 (hepatoma), J774A.1 (macrophage), B16F10 (melanoma), and primary mouse splenocytes, with different cell death inducers such as staurosporine (apoptosis), rapamycin (autophagy), erastin (ferroptosis), TNF-α (necroptosis), and nigericin (pyroptosis). Flow cytometry analysis showed a significant upregulation of surface PSGL-1 expression under all stress conditions across these cell types (Figure 1; example of flow cytometry gating is available in Figure S7), indicating that PSGL-1 acts as a universal marker for dying cells.

### 3.2. Increased Surface PSGL-1 Expression Is Associated with Apoptosis Induction in Hepatoma Huh-7 Cells

**Figure 2:**
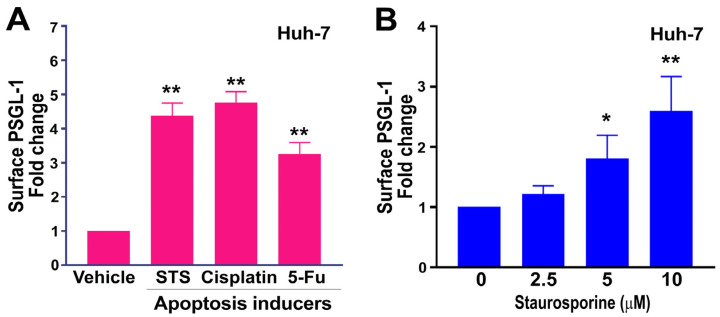
Apoptosis inducers induced surface PSGL-1 expression in Huh-7 cells. (**A**) Apoptosis inducers like staurosporine (STS; 10 μM), cisplatin (50 μM), and 5-FU (50 μM) increased surface PSGL-1 expression on Huh-7 cells after 4 h. (**B**) A dose-dependent increase in PSGL-1 expression on the surfaces of Huh-7 cells was observed with STS treatments ranging from 0 to 10 μM over 4 h. ** *p* < 0.01, * *p* < 0.05 vs. vehicle-treated groups (**A**,**B**). The vehicle groups (**A**,**B**) were set to 1-fold. Three experiments were performed with two replicates per group [*N* = 3 (3 independent experiments), with *n* = 2 (2 technical replicates per experiment)].

Because we plan to use TAA-induced hepatitis as an indication for in vivo evaluation of the effectiveness of this drug delivery system in a mouse model, the experiment was then focused on the analyses of hepatoma cell Huh-7. Apoptosis is one of the predominant cell death responses leading to upregulated surface PSGL-1 expression on the Huh-7 cells (Figure 1A). To see whether induction of surface PSGL-1 is a general effect induced by apoptosis inducers, different apoptosis inducers, which include staurosporine, cisplatin, and 5-Fu, were applied. Here we found that all tested apoptosis inducers induced marked upregulation of surface PSGL-1 expression on the Huh-7 cells (Figure 2A), suggesting that the induction of surface PSGL-1 is a general effect that could be applied to various apoptosis inducers. As our goal was to identify a surface marker expressed on the stressed cells and dying cells, here we wanted to further investigate whether cell-surface PSGL-1 expression is associated with the severity of apoptosis induction and whether we can use PSGL-1 as a cellular target to enable P-selectin-conjugated liposomes to more effectively bind and target these dying cells. Using a dose-dependent analysis, the results revealed that surface PSGL-1 expression (Figure 2B) was associated with the induction of apoptosis.

### 3.3. P-Selectin Is One of the Effective Proteins Enabling Conjugated Liposome Targeting to Apoptotic Huh-7 Cells

**Figure 3:**
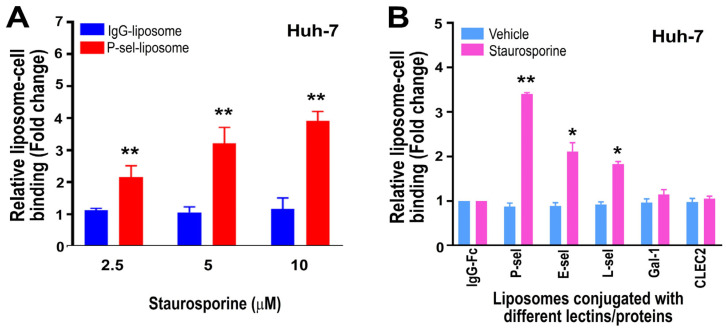
Huh-7 cell binding levels with fluorescent-dye-loaded liposomes were analyzed. Huh-7 cells were treated with or without the apoptosis inducer staurosporine, and their surface PSGL-1 expression (**A**) and engagement with fluorescein-loaded liposomes conjugated with various lectins and proteins (**B**) were measured using flow cytometry. Staurosporine treatment induced a dose-dependent increase in P-selectin (P-sel) liposome–Huh-7 cell interaction (**A**). ** *p* < 0.01 indicates a significant increase compared to the respective IgG-Fc–liposome groups (**A**). The IgG–liposome plus 2.5 μM STS-treated group (**A**) was set to 1-fold. (**B**) The conjugated lectins and proteins included IgG-Fc, P-selectin (P-sel), E-selectin (E-sel), L-selectin (L-sel), galectin-1 (Gal-1), and CLEC2. As these recombinant proteins contained an IgG-Fc portion, IgG-Fc was used as a control. (**B**) Both vehicle-treated and staurosporine-treated IgG-Fc groups were normalized to 1-fold. ** *p* < 0.01, * *p* < 0.05 indicates a significant increase compared to the respective IgG-Fc groups. The study comprised three experiments, with two replicates per group [*N* = 3 (3 independent experiments), *n* = 2 (2 technical replicates per experiment)].

We aimed to further investigate whether cell-surface PSGL-1 expression is correlated with P-selectin-conjugated liposome binding through dose-dependent analysis. In line with the cell-surface PSGL-1 expression results (Figure 2B), our findings showed that the interaction between Huh-7 cells and P-selectin-conjugated liposomes (P-sel-liposome; Figure 3A) was associated with apoptosis induction, whereas no such association was observed with IgG-Fc-conjugated liposomes (IgG-liposome; Figure 3A). PSGL-1 is known to bind and interact with multiple ligands, including E-selectin and L-selectin. To evaluate whether E-selectin and L-selectin more effectively target apoptotic hepatoma cells, we conducted cell–liposome binding experiments. Because P-selectin, E-selectin, and L-selectin used in the study were all IgG-Fc fusion recombinant proteins that contain an IgG-Fc region, an isotype-matched IgG-Fc served as the control protein. Furthermore, two lectins, galectin-1 and CLEC2, were included in the analysis for comparison to evaluate their potential similar effects. Additionally, to ensure that the liposomes were internalized by the cells rather than merely binding to the cell surface, NBD–liposomes were utilized. The results revealed that, compared to IgG-Fc-conjugated control liposomes, only P-selectin-conjugated liposomes displayed markedly enhanced binding ability to STS-treated apoptotic Huh-7 cells, surpassing E-selectin, L-selectin, galectin-1, and CLEC2-conjugated liposomes (Figure 3B; flow cytometry gating Figure S5).

### 3.4. Increased Surface PSGL-1 Expression and Enhanced Cell Engagement with P-Selectin-Conjugated Liposomes Were Observed in Dying ASGR1^+^ Mouse Hepatocytes Induced by RCD Inducers

**Figure 4:**
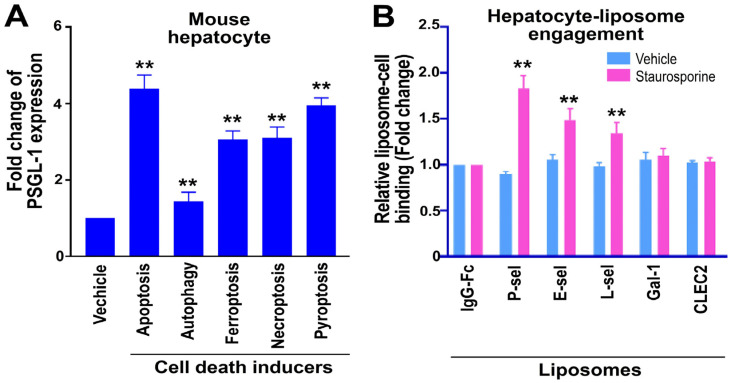
Surface PSGL-1 expression and engagement with liposomes conjugated to various lectins and proteins in primary ASGR1^+^ mouse hepatocytes. Primary ASGR1^+^ hepatocytes were treated with or without inducers of apoptosis (staurosporine, 10 μM), autophagy (rapamycin, 2 μM), ferroptosis (erastin, 20 μM), necroptosis (TNF-α, 0.2 μM), and pyroptosis (nigericin, 10 μM) for 4 h in vitro, followed by exposure to fluorescein-loaded liposomes conjugated with different lectins and proteins. Surface PSGL-1 expression levels (**A**) and cell–liposome engagement levels (**B**) were analyzed by flow cytometry. The conjugated lectins and proteins included IgG-Fc, P-sel, E-sel, L-sel, Gal-1, and CLEC2. Since these recombinant proteins all contained an IgG-Fc portion, an isotype-matched IgG-Fc was used as a control. Vehicle-treated groups (**A**) and IgG-Fc-treated hepatocyte groups (**B**) were normalized to 1-fold. ** *p* < 0.01 vs. vehicle-treated groups (**A**), ** *p* < 0.01 indicated a significant increase vs. respective IgG-Fc groups (**B**). The study was conducted with three independent experiments, each including two replicates per group [*N* = 3 (3 independent experiments), with *n* = 2 (2 technical replicates per experiment)].

Although we tested various lectin- and protein-conjugated liposomes on apoptotic Huh-7 hepatoma cells, the effect on normal hepatocytes remains unclear. To determine whether E-selectin and L-selectin more effectively target apoptotic ASGR1^+^ hepatocytes, we conducted cell–liposome binding experiments. Primary cell suspensions derived from mouse liver were treated with regulated cell death (RCD) inducers, including staurosporine (for apoptosis), rapamycin (for autophagy), erastin (for ferroptosis), TNF-α (for necroptosis), and nigericin (for pyroptosis), to evaluate surface PSGL-1 expression and interactions between cells and liposomes. After RCD inducer treatment, surface PSGL-1 expression levels in ASGR1^+^ hepatocytes were upregulated, and their interaction with P-selectin-conjugated liposomes increased compared to IgG-Fc-conjugated control liposomes. Among the tested liposomes—P-selectin, E-selectin, L-selectin, and control lectins (galectin-1 and CLEC2)—P-selectin-conjugated liposomes showed the strongest binding to staurosporine-treated apoptotic ASGR1^+^ hepatocytes (Figure 4; example of flow cytometry gating is available in Figure S6).

### 3.5. Apoptosis Is One of the Predominant RCD Responses of Mouse Hepatocytes After TAA Treatment

**Figure 5:**
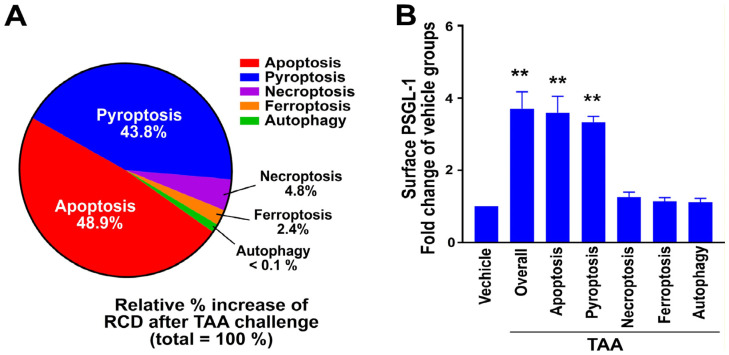
RCD profiling and PSGL-1 levels in hepatocytes from TAA-treated mice. (**A**) The RCD profile in ASGR1^+^ hepatocytes was assessed following TAA treatment using flow cytometry to evaluate changes in RCD levels. (**B**) Surface expression levels of PSGL-1 were measured in RCD-marker^+^ASGR1^+^ double-positive hepatocytes by flow cytometry. For example, in the apoptosis group, PSGL-1 levels were determined by identifying PSGL-1^+^ cells within the active caspase-3^+^ASGR1**^+^** double-positive population. The “overall” groups were analyzed for PSGL-1 expression in ASGR1^+^ hepatocytes, independent of RCD markers. Vehicle control groups were normalized to a 1-fold change. ** *p* < 0.01 vs. respective vehicle-treated groups. The study was conducted with three independent experiments, each including two replicates per group [*N* = 3 (3 independent experiments), with *n* = 2 (2 technical replicates per experiment)]. A total of 12 mice were used in the study.

To evaluate the effectiveness of drug delivery via the P-selectin–PSGL-1 axis in vivo, we used the TAA-induced hepatitis mouse model. Since a single cellular stressor can trigger multiple RCD responses simultaneously [56,57,58], we aimed to investigate the hepatocyte-specific RCD profile following TAA treatment. Using the hepatocyte-specific marker ASGR1, we identified that apoptosis and pyroptosis were the two predominant RCD responses, with apoptosis showing the greatest increase (Figure 5A; example of flow cytometry gating and the calculation of RCD profile is available in Figure S8). Thus, compared to other RCD pathways like necroptosis, ferroptosis, and autophagy, targeting apoptosis by loading an apoptosis inhibitor into P-sel liposomes may be more effective in rescuing TAA-induced hepatitis in mice. Additionally, consistent with in vitro findings, surface PSGL-1 expression was predominantly observed in apoptotic ASGR1^+^ hepatocytes after TAA treatment (Figure 5B).

### 3.6. Dying-Cell-Specific Targeting via the P-Selectin–PSGL-1 Axis Was Demonstrated by Fluorescent Dye Delivery to Injured Mouse Liver

**Figure 6:**
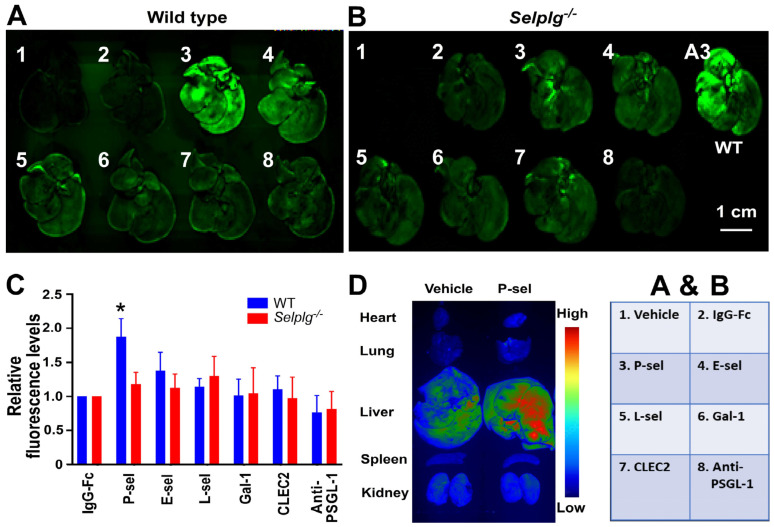
Dying-cell-specific targeting via the P-selectin–PSGL-1 axis demonstrated by fluorescent dye delivery to injured mouse liver. Following TAA-induced liver injury, wild-type (WT) (**A**) and PSGL-1 null (*Selplg*^−/−^) (**B**) mice were injected with fluorescein-loaded liposomes without protein conjugation (vehicle; 1), or conjugated to IgG-Fc (2), P-sel (3), E-sel (4), L-sel (5), Gal-1 (6), CLEC2 (7), and anti-PSGL-1 antibody (8). Fluorescent liver images are shown (A, B), and relative fluorescence intensities were quantified using ImageJ (**C**), in which the fluorescence levels of mouse liver in the IgG-Fc groups were normalized to 1-fold (**C**). (**D**) Pseudo-color imaging revealed that the system selectively delivered fluorescein to injured liver tissue while avoiding non-target organs like the lungs and spleen, where nanoparticles are typically retained. * *p* < 0.05 vs. IgG-Fc groups. Three experiments with two mice per group (*n* = 6). A total of 106 (53 WT, 53 *Selplg*^−/−^) mice were used in the study.

To assess the efficiency of this drug-delivery system, we employed fluorescein-loaded liposomes for quantification. Our results revealed that P-selectin is the most effective molecule conjugated to the liposome, facilitating the delivery of fluorescein into TAA-damaged mouse liver compared to other proteins like E-selectin, L-selectin, galectin-1, CLEC2, and anti-PSGL-1 Ig (Figure 6). Notably, this effect was observed only in wild-type mice, not in *Selplg*^−/−^ mice (Figure 6A–C), indicating a mechanism involving the P-selectin–PSGL-1 axis. Additionally, when examining other organs, we found that P-sel liposomes specifically targeted the liver while avoiding non-target organs such as the lung and spleen, where nanoparticles are usually retained (Figure 6D). This characteristic further underscores the potential of this P-selectin-based dying-cell delivery system.

### 3.7. TAA-Induced Liver Damage and Thrombocytopenia Were Markedly Ameliorated by P-Sel-Liposomes Loaded with Apoptosis Inhibitor z-DEVD via P-Selectin–PSGL-1 Axis

**Figure 7:**
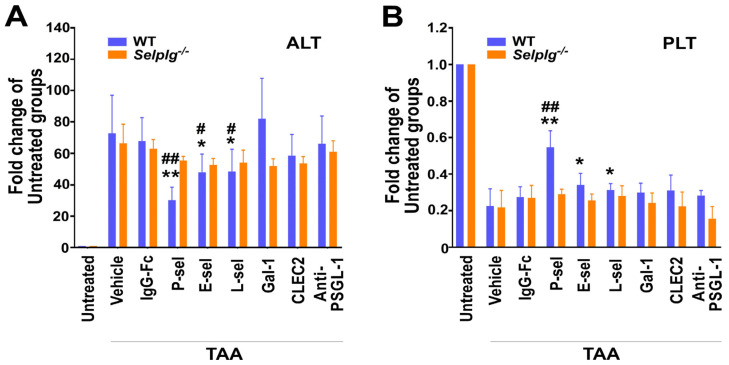
Rescue of damaged liver through dying-cell-specific targeting via the P-selectin–PSGL-1 axis with delivery of apoptosis inhibitor z-DEVD in mice. After TAA-induced liver injury, wild-type and *Selplg*^−/−^ mice were injected with caspase-3 inhibitor z-DEVD-loaded liposomes (5 × 10^7^ liposomes, containing 2 μM z-DEVD/mouse) conjugated to various proteins, including IgG-Fc, P-sel, E-sel, L-sel, Gal-1, CLEC2, and anti-PSGL-1 antibody (anti-PSGL-1). Plasma ALT levels (**A**) and platelet (PLT) counts (**B**) were measured. ** *p* < 0.01, * *p* < 0.05 vs. vehicle groups; ## *p* < 0.01, # *p* < 0.05 vs. IgG-Fc groups. Three experiments, with two mice per group (*n* = 6). A total of 108 (54 WT, 54 *Selplg*^−/−^) mice were used in the study.

We aimed to test whether the apoptosis caspase-3 inhibitor z-DEVD, loaded as cargo in this P-selectin-based dying cell delivery system, could rescue mice from TAA-induced liver damage, and associated pathogenesis. In previous studies, we have shown that TAA challenges lead to increased circulating ALT levels and hepatitis-associated thrombocytopenia in mice [26]. Accordingly, we assessed these two parameters in the current system. Our findings indicated that P-selectin is the most effective molecule conjugated to the liposome, facilitating rescue from TAA-induced increases in circulating ALT and thrombocytopenia, compared to other proteins such as E-selectin, L-selectin, galectin-1, CLEC2, and anti-PSGL-1 Ig (Figure 7). Notably, this effect was observed only in wild-type mice, not in *Selplg*^−/−^ mice (Figure 7), suggesting that the specific targeting of this delivery system is mediated through the P-selectin–PSGL-1 axis.

### 3.8. The Rescue Effect of Red-DEVD-Loaded P-Selectin Liposomes Is Facilitated by the P-Selectin–PSGL-1 Axis

**Figure 8:**
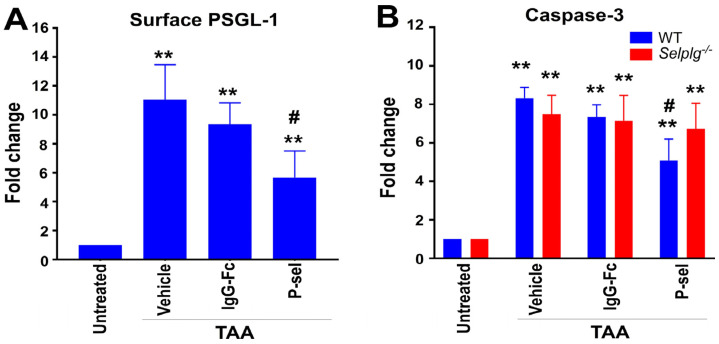
P-selectin-conjugated liposomes exert a rescue effect that is associated with the suppression of caspase-3 activities in mice. (**A**) Elevated surface PSGL-1 expression and (**B**) activated caspase-3 levels in TAA-treated mouse (wild type and *Selplg*^−/−^) hepatocytes in vivo were markedly rescued by treatments with P-selectin-conjugated liposomes (P-sel groups) loaded with caspase-3 inhibitor z-DEVD (5 × 10^7^ liposomes, containing 2 μM z-DEVD/mouse). The measurements of untreated groups were normalized to 1-fold. ** *p* < 0.01, indicated markedly exacerbated vs. respective untreated control groups; # *p* < 0.05 indicated markedly ameliorated vs. respective IgG-Fc control groups. Three experiments with two mice per group (*n* = 6) were performed. A total of 48 (24 WT, 24 *Selplg*^−/−^) mice were used in the study.

To further confirm that the z-DEVD-loaded P-selectin liposomes mitigate TAA-induced apoptosis-related liver abnormalities, we analyzed surface PSGL-1 and active caspase-3 in mouse ASGR1^+^ hepatocytes using flow cytometry. Results showed that the liposomes effectively reduced TAA-induced PSGL-1 expression (Figure 8A) and caspase-3 activation (Figure 8B), with the protective effect observed only in wild-type mice, not in *Selplg*^−/−^ mice, further indicating the involvement of PSGL-1.

**Figure 9:**
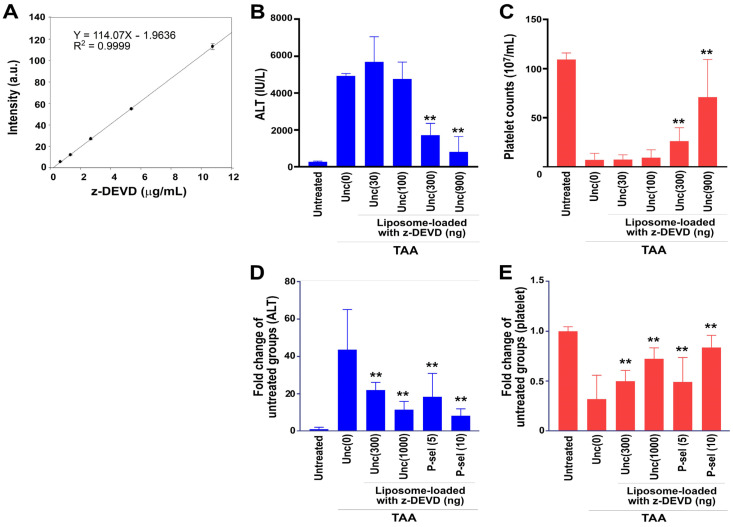
Markedly enhanced rescue efficiency was observed when Red-DEVD was loaded into P-selectin-conjugated liposomes in mice. (**A**) Quantification of Red-DEVD dosages was performed by measuring the fluorescence intensity of the fluorescent Red-DEVD. (**B**,**C**) In vivo testing of unconjugated liposomes (unc) loaded with Red-DEVD, with injection doses ranging from 30 ng to 900 ng per mouse [e.g., unc (30) represents unconjugated liposomes with 30 ng Red-DEVD], was performed to evaluate their ability to reduce TAA-induced increases in circulating ALT levels (**B**) and thrombocytopenia ((**C**), reduced platelet counts). The rescue effects on ALT levels (**D**) and thrombocytopenia (**E**) in TAA-treated mice, using unconjugated liposomes with Red-DEVD doses of 0–1000 ng per mouse, were compared to those using P-selectin-conjugated liposomes with Red-DEVD doses of 5–10 ng per mouse [e.g., P-sel (5) for P-selectin-conjugated liposomes with 5 ng Red-DEVD]. The untreated groups were normalized to 1-fold. ** *p* < 0.01 vs. respective TAA-treated unc (0) groups. Three experiments with two replicates per group (*n* = 6; (**A**–**C**)), and four experiments with three mice per group (*n* = 12; (**D**,**E**)) were conducted.

Next, we sought to evaluate the relative enhancement in drug-delivery efficiency offered by the P-selectin-based system in comparison to control liposomes without surface protein conjugation. We loaded various amounts of Red-DEVD into the control liposomes and treated both the control and P-selectin-conjugated liposomes with the fluorescent apoptosis inhibitor Red-DEVD. Initially, we measured the relative quantity of Red-DEVD using fluorescence intensity (Figure 9A). We then assessed the efficacy of protein-unconjugated liposomes loaded with Red-DEVD and discovered that administering 300–900 ng of Red-DEVD via unconjugated liposomes significantly alleviated the elevations in circulating ALT and thrombocytopenia in TAA-challenged mice (average mouse weight 28 g; Figure 9B,C).

Intriguingly, we observed that unconjugated liposomes required a 100-fold higher dose than P-selectin-conjugated liposomes (10 ng in P-selectin liposomes versus 1000 ng in unconjugated liposomes) to achieve a similar, but less effective, therapeutic outcome in reducing plasma ALT levels (Figure 9D) and alleviating thrombocytopenia (Figure 9E) in TAA-treated mice. This underscores that P-selectin conjugation increases drug delivery efficiency by at least 100-fold in TAA-challenged mice [Figure 9D,E, unc (1000) vs. P-sel (10)].

## 4. Discussion

Current knowledge and progress in cell and tissue-specific drug delivery systems are focused on improving the precision and efficiency of therapeutic targeting to diseased cells, such as cancer cells, while minimizing side effects on healthy tissues. Researchers are developing advanced technologies like nanoparticles, liposomes, and antibody–drug conjugates to enhance the targeting of specific tissues or cell types based on unique molecular markers [9,60]. These systems have shown promise in selectively delivering drugs to cancerous tissues, inflamed regions, or even specific organs like the liver or brain, enhancing treatment efficacy and reducing systemic toxicity [9,60]. However, these technologies face challenges, such as off-target effects and difficulties in effectively distinguishing between healthy and diseased cells [9,60].

A dying-cell-specific drug delivery system could address some of these limitations by focusing on cells undergoing regulated cell death (such as apoptosis), a process typically associated with disease or injury. By targeting unique markers like phosphatidylserine or PSGL-1 that are exposed on dying cells, this approach could enhance drug delivery specifically to tissues experiencing damage or disease-related cell death, thereby improving therapeutic outcomes. This strategy may complement existing technologies by providing an additional layer of precision, particularly in diseases where cell death plays a central role.

PSGL-1 is a critical molecule in the immune response, functioning as a ligand for selectins, a family of cell adhesion molecules that play a key role in mediating the interaction between leukocytes and the endothelial cells lining blood vessels [61,62]. PSGL-1 is expressed on the surface of various immune cells and binds to selectins under inflammatory conditions. This binding facilitates the rolling and tethering of leukocytes on the endothelium, a crucial step for their migration to sites of inflammation or injury [61,62]. The selectin family, which includes P-selectin, E-selectin, and L-selectin, plays a key role in initiating the inflammatory response by mediating cell–cell interactions critical for immune surveillance [63,64]. Unlike selectins, PSGL-1 is expressed more broadly across various cell types. In addition to its presence on leukocytes, it is also found on different cell types such as endothelial and stem cells [65,66,67]. Furthermore, PSGL-1 is recognized as a marker of cellular stress, including inflammation and exhaustion [68]. Therefore, we aimed to explore whether PSGL-1 can be targeted to deliver therapeutics specifically to stressed or dying cells in need of rescue.

In this study, we used a dying cell delivery system for the caspase-3 inhibitor z-DEVD and demonstrated its effectiveness in alleviating TAA-induced liver damage in mice. Caspase-3 inhibitors have been well documented for their use in animal models to address a wide range of diseases associated with cell death and tissue damage [69,70,71]. However, significant challenges remain for their clinical application [69,70].

The first caspase inhibitor to enter clinical trials was VX-740 (Pralnacasan), an orally administered pro-drug targeting caspase-1 [69]. Preclinical studies demonstrated its efficacy in reducing inflammation and arthritis in mice, and initial clinical trials in patients with rheumatoid arthritis and osteoarthritis confirmed its safety and effectiveness. However, the drug was eventually withdrawn due to liver toxicity observed in animal studies, despite no adverse effects being reported in human trials [70]. Similarly, GS-9450, an inhibitor of caspase-1, -8, and -9, showed potential in early trials for preventing liver damage but was discontinued following drug-induced liver injury in a larger study [69,70].

Due to these challenges, particularly concerning specificity and delivery, none of the caspase inhibitors have progressed beyond Phase II trials, despite their potential therapeutic benefits [69,70]. Additionally, the broad action of caspase inhibitors on cell death pathways complicates treatment, as unintended effects may occur. For example, z-VAD-FMK, a pan-caspase inhibitor, can inadvertently shift cell death from apoptosis to different RCD responses, potentially worsening inflammation and tissue damage [72,73,74,75]. This underscores the difficulty in controlling the timing, dosage, and specificity of caspase inhibitors, raising concerns about side effects like organ failure and aggravated inflammatory responses.

Caspases also play various roles unrelated to cell death, including cell growth, differentiation, and tumor suppression [76,77]. For caspase inhibitors to be viable in clinical settings, they must selectively target damaged or diseased cells without interfering with normal cell turnover, which is crucial for maintaining tissue homeostasis [76]. Consequently, developing advanced delivery systems, such as nanoparticle-based carriers, have been proposed as solutions for enhancing the safety and precision of these inhibitors [69,70]. Our findings indicate that using our dying cell delivery system allows for a reduction in z-DEVD usage to 1/100 of the standard dose (Figure 9), suggesting a significant decrease in potential adverse effects when administered to patients and highlighting a promising approach for using therapeutic agents with high specificity.

This dying-cell-specific drug delivery system offers distinct advantages over previously established methods. First, PSGL-1 was identified as a universal marker for dying cells, allowing for highly targeted drug delivery. Second, this specificity is further enhanced by the use of P-selectin-conjugated liposomes, which demonstrated superior binding efficiency to dying cells and effective delivery of therapeutic agents. Third, in vivo studies showed successful treatment of liver damage in wild-type mice, highlighting the system’s therapeutic potential. Fourth, unlike traditional nanoparticle or liposome-based delivery systems that often get trapped in the spleen and lungs [9,78], this system avoids such issues, ensuring more efficient and precise delivery to target tissues. These advancements suggest a promising approach for enhancing the safety and efficacy of targeted drug therapies.

Our findings showed that P-selectin conjugation significantly enhanced drug delivery efficiency compared to control liposomes [9,78]. As a result, a higher percentage of drug-loaded liposomes may therefore be more effective at reaching their dying cell targets. The exact mechanism underlying this enhanced efficiency requires further investigation. However, we have shown that soluble P-selectin provides protective benefits against various inflammatory conditions in mice [34,79], indicating that P-selectin may reduce leukocyte activation and prevent clearance via its anti-inflammatory effects. Furthermore, PSGL-1 has recently been recognized as a key player in immune checkpoint regulation, suppressing leukocyte activity [80,81]. In T cells, PSGL-1 negatively regulates immune responses, as demonstrated in a mouse model of inflammatory bowel disease where PSGL-1 deficiency worsened inflammation [82]. Similarly, in macrophages, PSGL-1 was found to maintain an immunosuppressed state [83]. These findings suggest that a drug delivery system leveraging the P-selectin–PSGL-1 axis may benefit from these immune-suppressing effects, reducing leukocyte clearance and enhancing targeting efficiency. Furthermore, our findings indicate that P-selectin, unlike other PSGL-1-binding proteins such as E-selectin, L-selectin, and anti-PSGL-1 Ig, may possess specific structural domains critical for high-efficiency delivery of dying cells, warranting further investigation.

Intriguingly, a recent study showed that targeting endothelial P-selectin can facilitate drug delivery across the blood–brain barrier [84]. While their system focuses on P-selectin as the target molecule on endothelial cells, our system targets PSGL-1 on dying cells, making the two approaches fundamentally different [84,85,86,87]. Despite this distinction, both systems demonstrate high efficiency in targeted drug delivery. This suggests that the P-selectin–PSGL-1 axis, beyond its established role in cell–cell adhesion, may also play a crucial role in the transport of materials across cell membranes and physiological barriers like the blood–brain barrier. Further investigation into this mechanism is warranted.

Chemical and drug-induced hepatitis occurs through mechanisms like oxidative stress, immune-mediated damage, and apoptosis, commonly triggered by substances such as acetaminophen and certain antibiotics. First-line treatments, such as N-acetylcysteine for liver injury and corticosteroids for immune-mediated damage, aim to mitigate injury but are nonspecific and do not directly target cellular processes [88,89,90,91]. Our dying-cell-specific drug delivery system, using P-selectin-conjugated liposomes loaded with the caspase-3 inhibitor z-DEVD, offers a targeted approach by selectively inhibiting apoptosis in damaged liver cells. This system could complement existing treatments by reducing further cell death in the liver without affecting healthy cells, thus minimizing off-target effects and enhancing therapeutic precision in managing liver damage.

In conclusion, our dying-cell-specific drug delivery system, utilizing P-selectin-conjugated liposomes loaded with the caspase-3 inhibitor z-DEVD, presents a novel and targeted approach to treating chemical and drug-induced liver damage by selectively inhibiting apoptosis in affected cells while sparing healthy tissue. This method could complement current treatments by enhancing therapeutic precision and minimizing off-target effects. However, limitations remain, such as optimizing dosing, understanding long-term safety, and ensuring that the system performs consistently across different types of liver injuries. Future perspectives should focus on refining this delivery system, exploring its efficacy in chronic liver diseases, and expanding its application to other organ systems to establish it as a versatile therapeutic platform.

## Data Availability

The datasets used and analyzed during the current study are available from the corresponding author on reasonable request.

## Supplementary Materials

The following supporting information can be downloaded at: https://www.mdpi.com/article/10.3390/cells13211778/s1, Figure S1: Measurement of protein conjugation efficiency. (A,B) Standard curves were generated by analyzing the relative protein concentrations of serially diluted IgG-Fc and P-selectin. (C) The efficiency of protein conjugation to liposomes was calculated using the formula Conjugation efficiency % = (Total − Free)/Total. The conjugation efficiency of IgG-Fc and P-selectin on liposomes was found to be approximately 70–75%.; Figure S2: Methods for quantifying liposome numbers. An example of flow cytometry gating illustrates 6 μm beads (A) alongside a mixture of 6 μm beads and liposomes (B). Using a known quantity of 6 μm beads (1 × 10^4^/mL) and calculating the ratio of beads (B region in panel B) to liposomes (A region in panel B), the number of liposomes can be quantified through flow cytometry analysis. Since the liposomes are smaller than 0.5 µm, the liposome and bead fractions can be easily separated and identified using forward scatter (FS; x-axis of the plots) and side scatter (SS; y-axis of the plots) analysis. Area A represents the liposome populations, while area B corresponds to the bead population. In panel B, beads accounted for 2.3% of the total population (gated number 581(B)/25254(All) = 2.3%; indicated by the red box). Given the concentration of beads is 1 × 10^4^/mL, the estimated concentration of liposomes in the A area of panel B was calculated to be 4.1 × 10^5^/mL; Figure S3: Measurement of liposome particle size. The particle size of the liposomes, including (A) control liposomes, (B) PEG-liposomes, (C) IgF-Fc-conjugated PEG-liposomes, and (D) P-selectin (P-sel)-conjugated PEG-liposomes, was determined using a particle size analyzer (ELSZ-2000, Otsuka). The average particle size of the major liposome population was approximately 1 μm in diameter (A–D). Signals exceeding 10 μm were due to liposome aggregations, which were significantly reduced by dilution; Figure S4: Measurement of encapsulation efficiency. (A) The relative fluorescence unit (RFU) was determined by measuring the fluorescence intensity across varying concentrations of fluorescein (30–150 μM). (B) The encapsulation efficiency (EE%) of various samples, including total fluorescein, fluorescein-loaded liposomes [Lipo(F)], fluorescein-loaded PEG-liposomes [PEG-lipo(F)], fluorescein-loaded IgG-Fc-conjugated PEG-liposomes [IgG-Fc-PEG-lipo(F)], and fluorescein-loaded P-selectin-conjugated PEG-liposomes [P-sel-PEG-lipo(F)], was calculated using the formula EE% = [(total drug added − free non-entrapped drug)/total drug added]. The encapsulation efficiency of fluorescein in both IgG-Fc- and P-selectin-conjugated liposomes was approximately 50%; Figure S5: Flow cytometry gating for measuring Huh-7 Cell engulfment of protein-conjugated NBD–liposomes. (A) Flow cytometry gating was applied to analyze vehicle-treated normal Huh-7 cells (A1-A3), staurosporine (STS)-treated apoptotic Huh-7 cells (A4-A6; STS-Huh-7), and STS-treated Huh-7 cells engaged with IgG-Fc-conjugated NBD–liposomes (A7-A9). Forward scatter (FSC) and side scatter (SSC) were used to distinguish Huh-7 cells from the smaller liposomes (A1, A4, A7), followed by gating of the apoptotic cell population using staining of active-form caspase-3 (A2, A5, A8) and identification of cells engulfed by NBD–liposomes (A3, A6, A9; green). Comparisons of different protein-conjugated liposomes, including IgG-Fc (B1), P-selectin (B2), E-selectin (B3), L-selectin (B4), galectin-1 (B5), and CLEC2 (B6), revealed that P-selectin-conjugated liposomes (B2) demonstrated the highest engulfment efficiency among all tested proteins. Note that A9 and B1 represent the same graph; Figure S6: An example illustrates how flow cytometry was used to determine the engagement levels of mouse ASGR1^+^ hepatocytes with Alexa Fluor 647-loaded liposomes. (A) The liver cell suspension was first gated using forward scatter and side scatter to identify liver cells, which were distinguishable from liposomes due to their larger size. (B) Mouse liver cells were further analyzed for ASGR1 expression, and only the ASGR1^+^ population was included in subsequent analyses. Cells were treated without (C–E, Normal groups) or with (F–H, Apo groups) the apoptosis inducer staurosporine, and the engagement of ASGR1^+^ hepatocytes with fluorescent dye Alexa Fluor 647 (A647)-loaded liposomes was assessed. Flow cytometry was used to detect A647 signals after ASGR1^+^ hepatocytes were exposed to vehicle (C,F), IgG-Fc-conjugated A647-loaded liposomes (D, G), and P-selectin-conjugated A647-loaded liposomes (E,H); Figure S7: An example of flow cytometry gating used to assess relative surface PSGL-1 expression in Huh-7 cells following treatment with various cell death inducers. The human hepatoma cell line Huh-7 was exposed to vehicle (A), apoptosis inducer (staurosporine) (B), autophagy inducer (rapamycin) (C), ferroptosis inducer (erastin) (D), necroptosis inducer (TNF-α) (E), and pyroptosis inducer (nigericin) (F) for 1 h. Cells were then double-stained with the Zombie NIR™ Fixable Viability Kit and a fluorescent-labeled anti-PSGL-1 antibody. PSGL-1 expression in the dying cell population was determined by identifying PSGL-1^+^ death signal^+^ double positive population in the upper-right quadrant of the flow cytometry panel; Figure S8: Calculation of the relative increase in regulated cell death (RCD) percentages of mouse liver hepatocytes following thioacetamide (TAA) treatment was performed using flow cytometry. Due to overlapping detection wavelengths, it is not feasible to detect all five RCD pathways (apoptosis, autophagy, ferroptosis, necroptosis, pyroptosis) simultaneously in a single staining sample. Thus, we conducted double staining for each RCD marker along with a live/dead cell status stain as an internal control. For example, hepatocytes isolated from vehicle-treated (A,C) or TAA-treated (B,D) mice were stained for specific RCD markers (A,B) and live/dead status (C,D). The increase in cell death signal (e.g., apoptosis signal B1-A1; ∆apoptosis) was normalized by the increased dead cell population (e.g., B1-A1/D1-C1; ∆apoptosis/∆total death cell) for each RCD. The sum of B1-A1/D1-C1, B2-A2/D2-C2, B3-A3/D3-C3, B4-A4/D4-C4, and B5-A5/D5-C5 was considered to be 100%; therefore, the relative apoptosis percentage was calculated as [B5-A5/D5-C5]/[(B1-A1/D1-C1) + (B2-A2/D2-C2) + (B3-A3/D3-C3) + (B4-A4/D4-C4) + (B5-A5/D5-C5)] × 100%, which was approximately 48.9%. The pie chart results shown in Figure 5A were generated using this formula, based on averaged data from triplicate samples per group.

## References

[1] Nkune N.W., Abrahamse H. (2021). Nanoparticle-Based Drug Delivery Systems for Photodynamic Therapy of Metastatic Melanoma: A Review. Int. J. Mol. Sci..

[2] Zhang Y., Almazi J.G., Ong H.X., Johansen M.D., Ledger S., Traini D., Hansbro P.M., Kelleher A.D., Ahlenstiel C.L. (2022). Nanoparticle Delivery Platforms for RNAi Therapeutics Targeting COVID-19 Disease in the Respiratory Tract. Int. J. Mol. Sci..

[3] Hersh A.M., Alomari S., Tyler B.M. (2022). Crossing the Blood-Brain Barrier: Advances in Nanoparticle Technology for Drug Delivery in Neuro-Oncology. Int. J. Mol. Sci..

[4] Markowski A., Jaromin A., Migdal P., Olczak E., Zygmunt A., Zaremba-Czogalla M., Pawlik K., Gubernator J. (2022). Design and Development of a New Type of Hybrid PLGA/Lipid Nanoparticle as an Ursolic Acid Delivery System against Pancreatic Ductal Adenocarcinoma Cells. Int. J. Mol. Sci..

[5] Nahrjou N., Ghosh A., Tanasova M. (2021). Targeting of GLUT5 for Transporter-Mediated Drug-Delivery Is Contingent upon Substrate Hydrophilicity. Int. J. Mol. Sci..

[6] Mitchell M.J., Billingsley M.M., Haley R.M., Wechsler M.E., Peppas N.A., Langer R. (2021). Engineering precision nanoparticles for drug delivery. Nat. Rev. Drug Discov..

[7] Liu G., Yang L., Chen G., Xu F., Yang F., Yu H., Li L., Dong X., Han J., Cao C. (2021). A Review on Drug Delivery System for Tumor Therapy. Front. Pharmacol..

[8] Srinivasarao M., Low P.S. (2017). Ligand-Targeted Drug Delivery. Chem. Rev..

[9] Zhao Z., Ukidve A., Kim J., Mitragotri S. (2020). Targeting Strategies for Tissue-Specific Drug Delivery. Cell.

[10] Peng H., He X., Wang Q. (2022). Targeted drug delivery system for ovarian cancer microenvironment: Improving the effects of immunotherapy. Front. Immunol..

[11] Cheng X., Xie Q., Sun Y. (2023). Advances in nanomaterial-based targeted drug delivery systems. Front. Bioeng. Biotechnol..

[12] Zhang J., Wang S., Zhang D., He X., Wang X., Han H., Qin Y. (2023). Nanoparticle-based drug delivery systems to enhance cancer immunotherapy in solid tumors. Front. Immunol..

[13] Yu L., Liu S., Jia S., Xu F. (2023). Emerging frontiers in drug delivery with special focus on novel techniques for targeted therapies. Biomed. Pharmacother..

[14] Liu X., Cheng Y., Mu Y., Zhang Z., Tian D., Liu Y., Hu X., Wen T. (2024). Diverse drug delivery systems for the enhancement of cancer immunotherapy: An overview. Front. Immunol..

[15] Wickline S.A., Hou K.K., Pan H. (2023). Peptide-Based Nanoparticles for Systemic Extrahepatic Delivery of Therapeutic Nucleotides. Int. J. Mol. Sci..

[16] Del Genio V., Falanga A., Allard-Vannier E., Herve-Aubert K., Leone M., Bellavita R., Uzbekov R., Chourpa I., Galdiero S. (2022). Design and Validation of Nanofibers Made of Self-Assembled Peptides to Become Multifunctional Stimuli-Sensitive Nanovectors of Anticancer Drug Doxorubicin. Pharmaceutics.

[17] Jakubowska E., Milanowski B., Lulek J. (2021). A Systematic Approach to the Development of Cilostazol Nanosuspension by Liquid Antisolvent Precipitation (LASP) and Its Combination with Ultrasound. Int. J. Mol. Sci..

[18] Egorova E.A., Nikitin M.P. (2022). Delivery of Theranostic Nanoparticles to Various Cancers by Means of Integrin-Binding Peptides. Int. J. Mol. Sci..

[19] Vaneev A., Tikhomirova V., Chesnokova N., Popova E., Beznos O., Kost O., Klyachko N. (2021). Nanotechnology for Topical Drug Delivery to the Anterior Segment of the Eye. Int. J. Mol. Sci..

[20] Tang D., Kang R., Berghe T.V., Vandenabeele P., Kroemer G. (2019). The molecular machinery of regulated cell death. Cell Res..

[21] Ai Y., Meng Y., Yan B., Zhou Q., Wang X. (2024). The biochemical pathways of apoptotic, necroptotic, pyroptotic, and ferroptotic cell death. Mol. Cell.

[22] Vitale I., Pietrocola F., Guilbaud E., Aaronson S.A., Abrams J.M., Adam D., Agostini M., Agostinis P., Alnemri E.S., Altucci L. (2023). Apoptotic cell death in disease-Current understanding of the NCCD 2023. Cell Death Differ..

[23] Sepehrinezhad A., Shahbazi A., Sahab Negah S., Joghataei M.T., Larsen F.S. (2021). Drug-induced-acute liver failure: A critical appraisal of the thioacetamide model for the study of hepatic encephalopathy. Toxicol. Rep..

[24] Shin M.R., Lee J.A., Kim M., Lee S., Oh M., Moon J., Nam J.W., Choi H., Mun Y.J., Roh S.S. (2021). Gardeniae Fructus Attenuates Thioacetamide-Induced Liver Fibrosis in Mice via Both AMPK/SIRT1/NF-kappaB Pathway and Nrf2 Signaling. Antioxidants.

[25] ElBaset M.A., Salem R.S., Ayman F., Ayman N., Shaban N., Afifi S.M., Esatbeyoglu T., Abdelaziz M., Elalfy Z.S. (2022). Effect of Empagliflozin on Thioacetamide-Induced Liver Injury in Rats: Role of AMPK/SIRT-1/HIF-1alpha Pathway in Halting Liver Fibrosis. Antioxidants.

[26] Lin Y.Y., Hu C.T., Sun D.S., Lien T.S., Chang H.H. (2019). Thioacetamide-induced liver damage and thrombocytopenia is associated with induction of antiplatelet autoantibody in mice. Sci. Rep..

[27] Okuyama H., Shimahara Y., Nakamura H., Araya S., Kawada N., Yamaoka Y., Yodoi J. (2004). Thioredoxin prevents thioacetamide-induced acute hepatitis. Comp. Hepatol..

[28] Miyata T., Nagy L.E. (2020). Programmed cell death in alcohol-associated liver disease. Clin. Mol. Hepatol..

[29] Iorga A., Dara L. (2019). Cell death in drug-induced liver injury. Adv. Pharmacol..

[30] Iorga A., Dara L., Kaplowitz N. (2017). Drug-Induced Liver Injury: Cascade of Events Leading to Cell Death, Apoptosis or Necrosis. Int. J. Mol. Sci..

[31] Peters D.T., Henderson C.A., Warren C.R., Friesen M., Xia F., Becker C.E., Musunuru K., Cowan C.A. (2016). Asialoglycoprotein receptor 1 is a specific cell-surface marker for isolating hepatocytes derived from human pluripotent stem cells. Development.

[32] Ashwell G., Morell A.G. (1974). The role of surface carbohydrates in the hepatic recognition and transport of circulating glycoproteins. Adv. Enzymol. Relat. Areas Mol. Biol..

[33] Schwartz A.L., Marshak-Rothstein A., Rup D., Lodish H.F. (1981). Identification and quantification of the rat hepatocyte asialoglycoprotein receptor. Proc. Natl. Acad. Sci. USA.

[34] Sun D.S., Chang Y.W., Kau J.H., Huang H.H., Ho P.H., Tzeng Y.J., Chang H.H. (2017). Soluble P-selectin rescues mice from anthrax lethal toxin-induced mortality through PSGL-1 pathway-mediated correction of hemostasis. Virulence.

[35] Li C.C., Munalisa R., Lee H.Y., Lien T.S., Chan H., Hung S.C., Sun D.S., Cheng C.F., Chang H.H. (2023). Restraint Stress-Induced Immunosuppression Is Associated with Concurrent Macrophage Pyroptosis Cell Death in Mice. Int. J. Mol. Sci..

[36] Munalisa R., Lien T.S., Tsai P.Y., Sun D.S., Cheng C.F., Wu W.S., Li C.C., Hu C.T., Tsai K.W., Lee Y.L. (2024). Restraint Stress-Induced Neutrophil Inflammation Contributes to Concurrent Gastrointestinal Injury in Mice. Int. J. Mol. Sci..

[37] Feng M., Divall S., Wu S. (2021). An Improved Time- and Labor- Efficient Protocol for Mouse Primary Hepatocyte Isolation. J. Vis. Exp..

[38] Charni-Natan M., Goldstein I. (2020). Protocol for Primary Mouse Hepatocyte Isolation. STAR Protoc..

[39] Silva V.R., Correa R.S., Santos L.S., Soares M.B.P., Batista A.A., Bezerra D.P. (2018). A ruthenium-based 5-fluorouracil complex with enhanced cytotoxicity and apoptosis induction action in HCT116 cells. Sci. Rep..

[40] Shen X., Wang H., Weng C., Jiang H., Chen J. (2021). Caspase 3/GSDME-dependent pyroptosis contributes to chemotherapy drug-induced nephrotoxicity. Cell Death Dis..

[41] Mao X., Li J., Xie X., Chen S., Huang Q., Mu P., Jiang J., Deng Y. (2022). Deoxynivalenol induces caspase-3/GSDME-dependent pyroptosis and inflammation in mouse liver and HepaRG cells. Arch. Toxicol..

[42] Sun D.S., Lee P.C., Kau J.H., Shih Y.L., Huang H.H., Li C.R., Lee C.C., Wu Y.P., Chen K.C., Chang H.H. (2015). Acquired coagulant factor VIII deficiency induced by Bacillus anthracis lethal toxin in mice. Virulence.

[43] Chang H.H., Chang C.P., Chang J.C., Dung S.Z., Lo S.J. (1997). Application of Recombinant Rhodostomin in Studying Cell Adhesion. J. Biomed. Sci..

[44] Feng G., Kaplowitz N. (2002). Mechanism of staurosporine-induced apoptosis in murine hepatocytes. Am. J. Physiol. Gastrointest. Liver Physiol..

[45] Harley W., Floyd C., Dunn T., Zhang X.D., Chen T.Y., Hegde M., Palandoken H., Nantz M.H., Leon L., Carraway K.L. (2010). Dual inhibition of sodium-mediated proton and calcium efflux triggers non-apoptotic cell death in malignant gliomas. Brain Res..

[46] Fu J., Yu M., Xu W., Yu S. (2023). High Expression of G9a Induces Cisplatin Resistance in Hepatocellular Carcinoma. Cell J..

[47] Kim Y., Jang M., Lim S., Won H., Yoon K.S., Park J.H., Kim H.J., Kim B.H., Park W.S., Ha J. (2011). Role of cyclophilin B in tumorigenesis and cisplatin resistance in hepatocellular carcinoma in humans. Hepatology.

[48] Brenes O., Arce F., Gatjens-Boniche O., Diaz C. (2007). Characterization of cell death events induced by anti-neoplastic drugs cisplatin, paclitaxel and 5-fluorouracil on human hepatoma cell lines: Possible mechanisms of cell resistance. Biomed. Pharmacother..

[49] Kwon S., Jeon J.S., Ahn C., Sung J.S., Choi I. (2016). Rapamycin regulates the proliferation of Huh7, a hepatocellular carcinoma cell line, by up-regulating p53 expression. Biochem. Biophys. Res. Commun..

[50] Fischer T.D., Wang J.H., Vlada A., Kim J.S., Behrns K.E. (2014). Role of autophagy in differential sensitivity of hepatocarcinoma cells to sorafenib. World J. Hepatol..

[51] Zheng J., Sato M., Mishima E., Sato H., Proneth B., Conrad M. (2021). Sorafenib fails to trigger ferroptosis across a wide range of cancer cell lines. Cell Death Dis..

[52] Salama M.F., Bayele H.K., Srai S.S. (2012). Tumour necrosis factor alpha downregulates human hemojuvelin expression via a novel response element within its promoter. J. Biomed. Sci..

[53] Zhao X., Cao M., Liu J.J., Zhu H., Nelson D.R., Liu C. (2011). Reactive oxygen species is essential for cycloheximide to sensitize lexatumumab-induced apoptosis in hepatocellular carcinoma cells. PLoS ONE.

[54] Daussy C.F., Monard S.C., Guy C., Munoz-Gonzalez S., Chazal M., Anthonsen M.W., Jouvenet N., Henry T., Dreux M., Meurs E.F. (2021). The Inflammasome Components NLRP3 and ASC Act in Concert with IRGM To Rearrange the Golgi Apparatus during Hepatitis C Virus Infection. J. Virol..

[55] Wu L., Bai S., Huang J., Cui G., Li Q., Wang J., Du X., Fu W., Li C., Wei W. (2023). Nigericin Boosts Anti-Tumor Immune Response via Inducing Pyroptosis in Triple-Negative Breast Cancer. Cancers.

[56] Lien T.S., Sun D.S., Wu C.Y., Chang H.H. (2021). Exposure to Dengue Envelope Protein Domain III Induces Nlrp3 Inflammasome-Dependent Endothelial Dysfunction and Hemorrhage in Mice. Front. Immunol..

[57] Lien T.S., Chan H., Sun D.S., Wu J.C., Lin Y.Y., Lin G.L., Chang H.H. (2021). Exposure of Platelets to Dengue Virus and Envelope Protein Domain III Induces Nlrp3 Inflammasome-Dependent Platelet Cell Death and Thrombocytopenia in Mice. Front. Immunol..

[58] Lien T.S., Sun D.S., Hung S.C., Wu W.S., Chang H.H. (2021). Dengue Virus Envelope Protein Domain III Induces Nlrp3 Inflammasome-Dependent NETosis-Mediated Inflammation in Mice. Front. Immunol..

[59] Hung S.C., Ke L.C., Lien T.S., Huang H.S., Sun D.S., Cheng C.L., Chang H.H. (2022). Nanodiamond-Induced Thrombocytopenia in Mice Involve P-Selectin-Dependent Nlrp3 Inflammasome-Mediated Platelet Aggregation, Pyroptosis and Apoptosis. Front. Immunol..

[60] Ezike T.C., Okpala U.S., Onoja U.L., Nwike C.P., Ezeako E.C., Okpara O.J., Okoroafor C.C., Eze S.C., Kalu O.L., Odoh E.C. (2023). Advances in drug delivery systems, challenges and future directions. Heliyon.

[61] Escopy S., Chaikof E.L. (2024). Targeting the P-selectin/PSGL-1 pathway: Discovery of disease-modifying therapeutics for disorders of thromboinflammation. Blood Vessel. Thromb. Hemost..

[62] Patel M.S., Miranda-Nieves D., Chen J., Haller C.A., Chaikof E.L. (2017). Targeting P-selectin glycoprotein ligand-1/P-selectin interactions as a novel therapy for metabolic syndrome. Transl. Res..

[63] Somers W.S., Tang J., Shaw G.D., Camphausen R.T. (2000). Insights into the molecular basis of leukocyte tethering and rolling revealed by structures of P- and E-selectin bound to SLe(X) and PSGL-1. Cell.

[64] Ivetic A., Hoskins Green H.L., Hart S.J. (2019). L-selectin: A Major Regulator of Leukocyte Adhesion, Migration and Signaling. Front. Immunol..

[65] Zhao X., Liu H.Q., Li J., Liu X.L. (2016). Endothelial progenitor cells promote tumor growth and progression by enhancing new vessel formation. Oncol. Lett..

[66] Wu X., Liu X., Yang H., Chen Q., Zhang N., Li Y., Du X., Liu X., Jiang X., Jiang Y. (2022). P-Selectin Glycoprotein Ligand-1 Deficiency Protects Against Aortic Aneurysm Formation Induced by DOCA Plus Salt. Cardiovasc. Drugs Ther..

[67] da Costa Martins P., Garcia-Vallejo J.J., van Thienen J.V., Fernandez-Borja M., van Gils J.M., Beckers C., Horrevoets A.J., Hordijk P.L., Zwaginga J.J. (2007). P-selectin glycoprotein ligand-1 is expressed on endothelial cells and mediates monocyte adhesion to activated endothelium. Arterioscler. Thromb. Vasc. Biol..

[68] Zaongo S.D., Chen Y. (2023). PSGL-1, a Strategic Biomarker for Pathological Conditions in HIV Infection: A Hypothesis Review. Viruses.

[69] Kudelova J., Fleischmannova J., Adamova E., Matalova E. (2015). Pharmacological caspase inhibitors: Research towards therapeutic perspectives. J. Physiol. Pharmacol..

[70] Dhani S., Zhao Y., Zhivotovsky B. (2021). A long way to go: Caspase inhibitors in clinical use. Cell Death Dis..

[71] Khan S., Ahmad K., Alshammari E.M., Adnan M., Baig M.H., Lohani M., Somvanshi P., Haque S. (2015). Implication of Caspase-3 as a Common Therapeutic Target for Multineurodegenerative Disorders and Its Inhibition Using Nonpeptidyl Natural Compounds. Biomed. Res. Int..

[72] Wu Y.T., Tan H.L., Huang Q., Sun X.J., Zhu X., Shen H.M. (2011). zVAD-induced necroptosis in L929 cells depends on autocrine production of TNFalpha mediated by the PKC-MAPKs-AP-1 pathway. Cell Death Differ..

[73] Sakthivel D., Bolivar B.E., Bouchier-Hayes L. (2022). Cellular autophagy, an unbidden effect of caspase inhibition by zVAD-fmk. FEBS J..

[74] Cauwels A., Janssen B., Waeytens A., Cuvelier C., Brouckaert P. (2003). Caspase inhibition causes hyperacute tumor necrosis factor-induced shock via oxidative stress and phospholipase A2. Nat. Immunol..

[75] Vandenabeele P., Vanden Berghe T., Festjens N. (2006). Caspase inhibitors promote alternative cell death pathways. Sci. STKE.

[76] Eskandari E., Eaves C.J. (2022). Paradoxical roles of caspase-3 in regulating cell survival, proliferation, and tumorigenesis. J. Cell Biol..

[77] Shalini S., Dorstyn L., Dawar S., Kumar S. (2015). Old, new and emerging functions of caspases. Cell Death Differ..

[78] Vargason A.M., Anselmo A.C., Mitragotri S. (2021). The evolution of commercial drug delivery technologies. Nat. Biomed. Eng..

[79] Sun D.S., Ho P.H., Chang H.H. (2016). Soluble P-selectin rescues viper venom-induced mortality through anti-inflammatory properties and PSGL-1 pathway-mediated correction of hemostasis. Sci. Rep..

[80] Tinoco R., Otero D.C., Takahashi A.A., Bradley L.M. (2017). PSGL-1: A New Player in the Immune Checkpoint Landscape. Trends Immunol..

[81] DeRogatis J.M., Viramontes K.M., Neubert E.N., Henriquez M.L., Guerrero-Juarez C.F., Tinoco R. (2022). Targeting the PSGL-1 Immune Checkpoint Promotes Immunity to PD-1-Resistant Melanoma. Cancer Immunol. Res..

[82] Matsumoto M., Miyasaka M., Hirata T. (2009). P-selectin glycoprotein ligand-1 negatively regulates T-cell immune responses. J. Immunol..

[83] Kauffman K., Manfra D., Nowakowska D., Zafari M., Nguyen P.A., Phennicie R., Vollmann E.H., O’Nuallain B., Basinski S., Komoroski V. (2023). PSGL-1 Blockade Induces Classical Activation of Human Tumor-associated Macrophages. Cancer Res. Commun..

[84] Tylawsky D.E., Kiguchi H., Vaynshteyn J., Gerwin J., Shah J., Islam T., Boyer J.A., Boue D.R., Snuderl M., Greenblatt M.B. (2023). P-selectin-targeted nanocarriers induce active crossing of the blood-brain barrier via caveolin-1-dependent transcytosis. Nat. Mater..

[85] Wang L., Wilhelm S. (2023). Exploiting endothelial transcytosis to reach into the brain. Nat. Mater..

[86] Chang H.H., Sun D.S. (2020). Vesicles comprising lectins expressed on the surface and methods of use thereof to deliver an agent to autophagic and apoptotic cells. USA patent.

[87] Chang H.H., Sun D.S. (2022). Delivery of agents to autophagy and apoptotic cells via vesicles having proteins expressed on their surfaces. China patent.

[88] Walayat S., Shoaib H., Asghar M., Kim M., Dhillon S. (2021). Role of N-acetylcysteine in non-acetaminophen-related acute liver failure: An updated meta-analysis and systematic review. Ann. Gastroenterol..

[89] Sanabria-Cabrera J., Tabbai S., Niu H., Alvarez-Alvarez I., Licata A., Bjornsson E., Andrade R.J., Lucena M.I. (2022). N-Acetylcysteine for the Management of Non-Acetaminophen Drug-Induced Liver Injury in Adults: A Systematic Review. Front. Pharmacol..

[90] Ntamo Y., Ziqubu K., Chellan N., Nkambule B.B., Nyambuya T.M., Mazibuko-Mbeje S.E., Gabuza K.B., Marcheggiani F., Tiano L., Dludla P.V. (2021). Drug-Induced Liver Injury: Clinical Evidence of N-Acetyl Cysteine Protective Effects. Oxidative Med. Cell. Longev..

[91] Bjornsson E.S., Vucic V., Stirnimann G., Robles-Diaz M. (2022). Role of Corticosteroids in Drug-Induced Liver Injury. A Systematic Review. Front. Pharmacol..

