# Supplementary material for: Targeted Delivery to Dying Cells Through P-Selectin–PSGL-1 Axis: A Promising Strategy for Enhanced Drug Efficacy in Liver Injury Models
Source: Cells. 2024 Oct 27;13(21):1778. doi: 10.3390/cells13211778 (PMC11545035; doi:10.3390/cells13211778)
Supplement: Supplementary file 1 [file cells-13-01778-s001.zip › cells-3263967-supplementary.pdf]

Supplemental information for:

Targeted Delivery to Dying Cells through P-Selectin-PSGL-1 Axis: A Promising Strategy for Enhanced Drug Efficacy in Liver Injury Models

Te-Sheng Lien, Der-Shan Sun, and Hsin-Hou Chang*

Department of Molecular Biology and Human Genetics, Tzu-Chi University, Hualien 970, Taiwan.

* To whom correspondence and reprint requests should be addressed.
Hsin-Hou Chang Ph.D.
Room D407, Tzu-Chi University, No. 701, Section 3, Chung-Yang Road, Hualien 97004, Taiwan.
Tel: 886-3-8565301 ext 2667. Fax: 886-3-8578386.
E-mail: hhchang@mail.tcu.edu.tw

Figure S1.

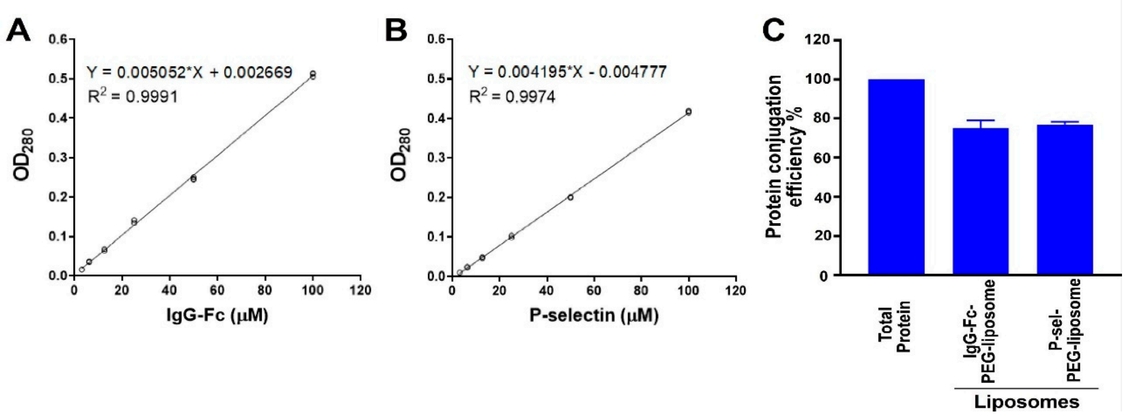

Figure S1. Measurement of protein conjugation efficiency. (A and B) Standard curves were generated by analyzing the relative protein concentrations of serially diluted IgG-Fc and P-selectin. (C) The efficiency of protein conjugation to liposomes was calculated using the formula: Conjugation efficiency % = (Total – Free) / Total. The conjugation efficiency of IgG-Fc and P-selectin on liposomes was found to be approximately 70-75%.

Figure S2.

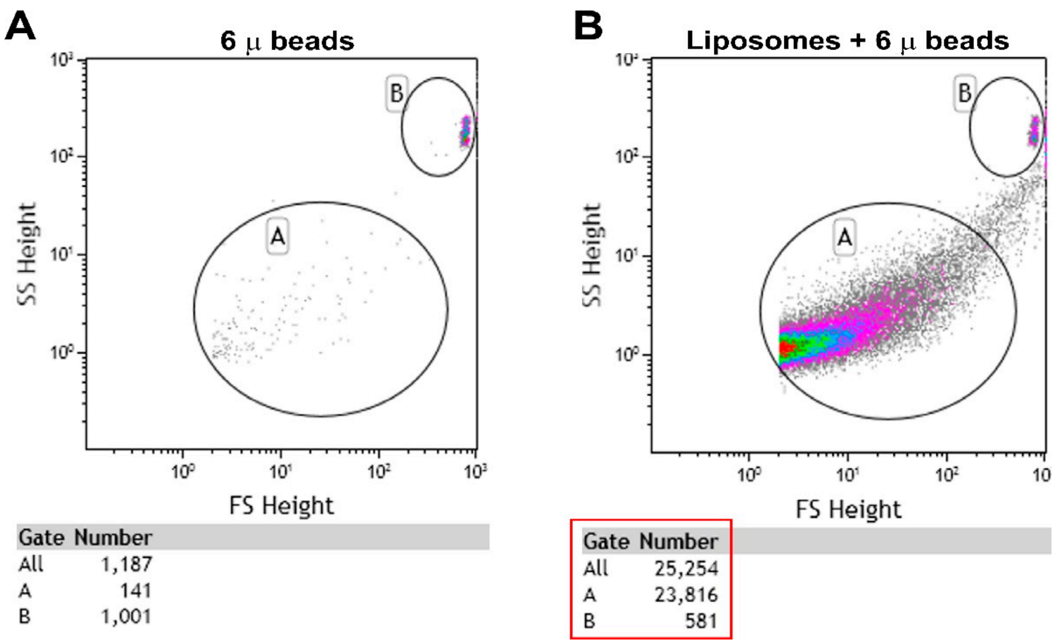

Figure S2. Methods for quantifying liposome numbers. An example of flow cytometry gating illustrates 6 μm beads (A) alongside a mixture of 6 μm beads and liposomes (B). Using a known quantity of 6 μm beads (1×10^4/mL) and calculating the ratio of beads (B region in panel B) to liposomes (A region in panel B), the number of liposomes can be quantified through flow cytometry analysis. Since the liposomes are smaller than 0.5 μm, the liposome and bead fractions can be easily separated and identified using forward scatter (FS; x-axis of the plots) and side scatter (SS; y-axis of the plots) analysis. Area A represents the liposome populations, while area B corresponds to the bead population. In panel B, beads accounted for 2.3% of the total population (gated number 581(B)/25254(All) = 2.3%; indicated by the red box). Given the concentration of beads is 1×10^4/mL, the estimated concentration of liposomes in the A area of panel B was calculated to be 4.1×10^5/mL.

Figure S3.

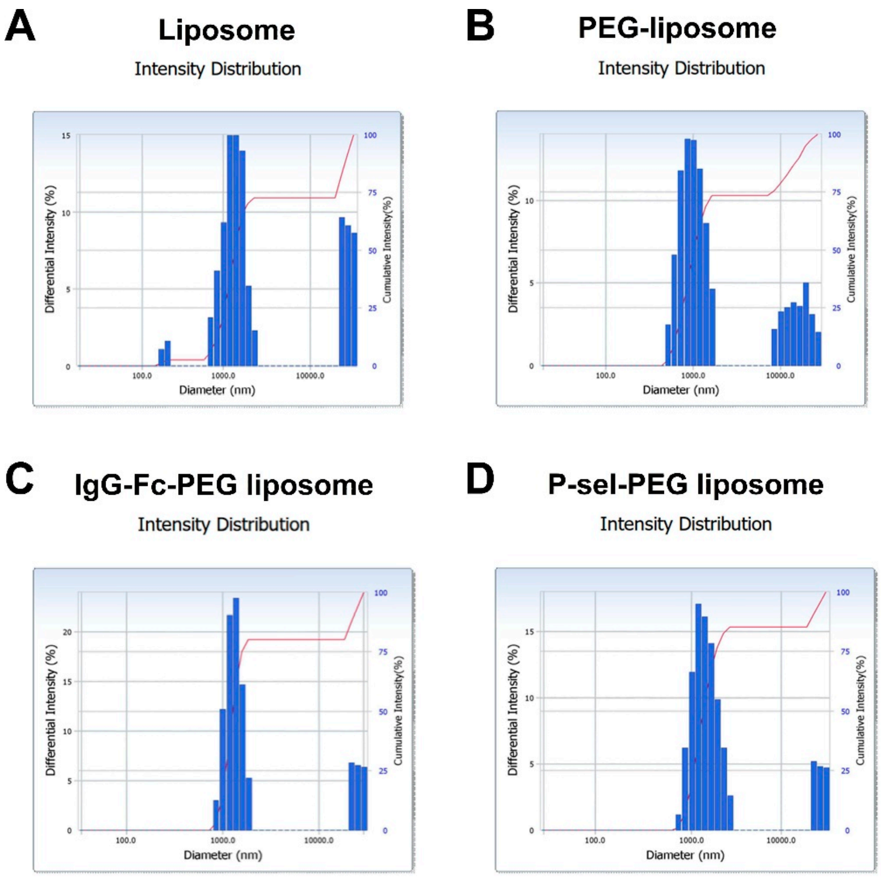

Figure S3. Measurement of liposome particle size. The particle size of the liposomes, including (A) control liposomes, (B) PEG-liposomes, (C) IgF-Fc-conjugated PEG-liposomes, and (D) P-selectin (P-sel)-conjugated PEG-liposomes, was determined using a particle size analyzer (ELSZ-2000, Otsuka). The average particle size of the major liposome population was approximately 1 μm in diameter (A-D). Signals exceeding 10 μm were due to liposome aggregations, which were significantly reduced by dilution.

Figure S4.

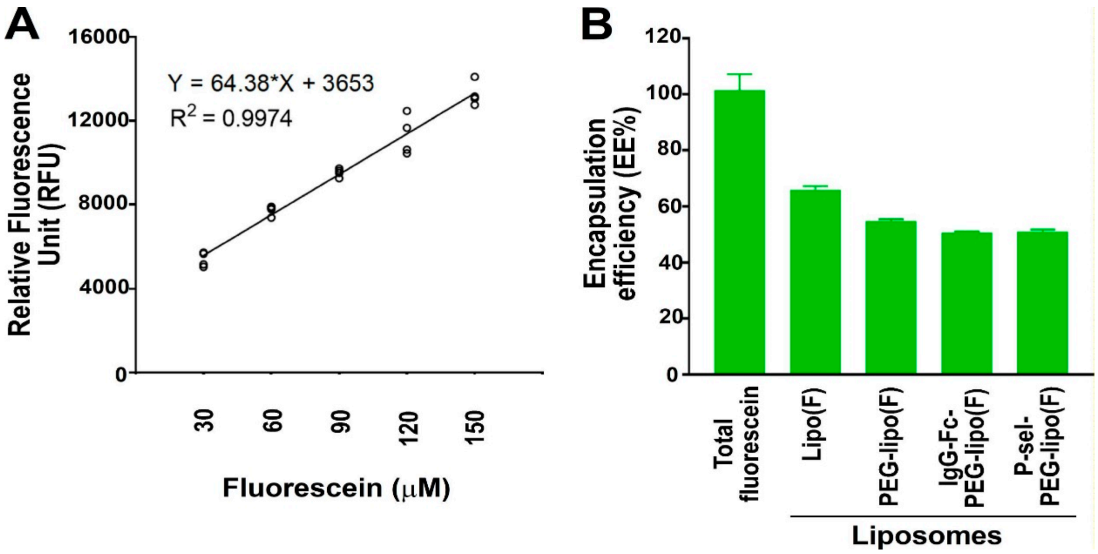

Figure S4. Measurement of encapsulation efficiency. (A) The relative fluorescence unit (RFU) was determined by measuring the fluorescence intensity across varying concentrations of fluorescein (30-150 μM). (B) The encapsulation efficiency (EE%) of various samples, including total fluorescein, fluorescein-loaded liposomes [Lipo(F)], fluorescein-loaded PEG-liposomes [PEG-lipo(F)], fluorescein-loaded IgG-Fc-conjugated PEG-liposomes [IgG-Fc-PEG-lipo(F)], and fluorescein-loaded P-selectin-conjugated PEG-liposomes [P-sel-PEG-lipo(F)], was calculated using the formula: EE% = [(total drug added – free non-entrapped drug) / total drug added]. The encapsulation efficiency of fluorescein in both IgG-Fc- and P-selectin-conjugated liposomes was approximately 50%.

Figure S5.

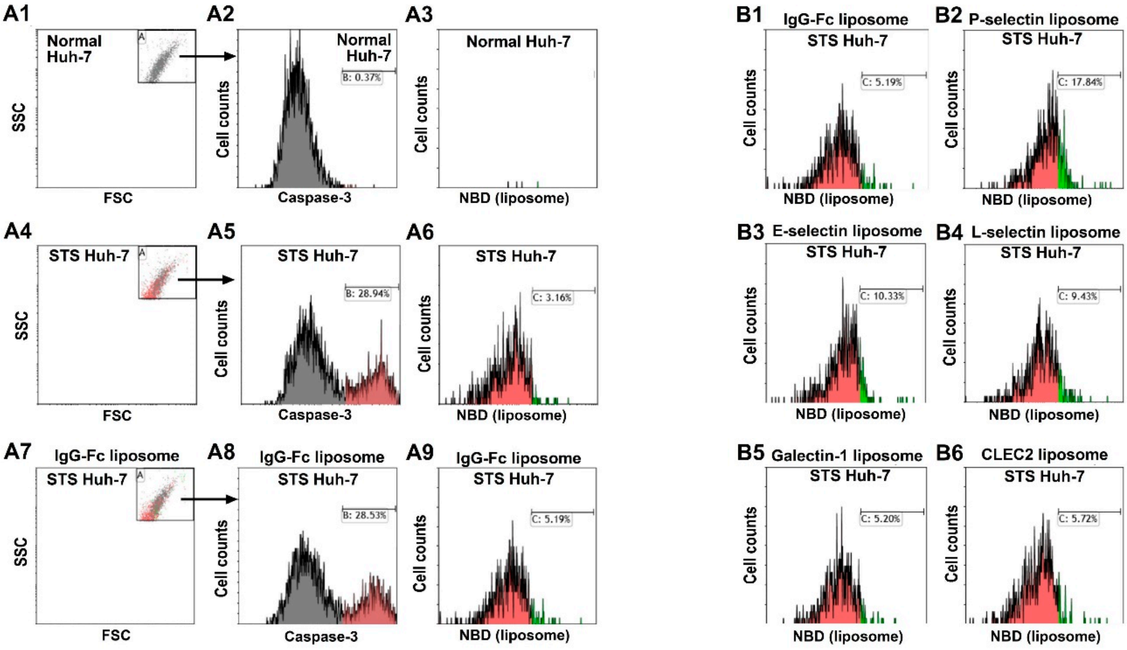

Figure S5. Flow cytometry gating for measuring Huh-7 Cell engulfment of protein-conjugated NBD-liposomes. (A) Flow cytometry gating was applied to analyze vehicle-treated normal Huh-7 cells (A1-A3), staurosporine (STS)-treated apoptotic Huh-7 cells (A4-A6; STS-Huh-7), and STS-treated Huh-7 cells engaged with IgG-Fc-conjugated NBD-liposomes (A7-A9). Forward scatter (FSC) and side scatter (SSC) were used to distinguish Huh-7 cells from the smaller liposomes (A1, A4, A7), followed by gating of the apoptotic cell population using staining of active-form caspase-3 (A2, A5, A8), and identification of cells engulfed by NBD-liposomes (A3, A6, A9; green). Comparisons of different protein-conjugated liposomes, including IgG-Fc (B1), P-selectin (B2), E-selectin (B3), L-selectin (B4), galectin-1 (B5), and CLEC2 (B6), revealed that P-selectin-conjugated liposomes (B2) demonstrated the highest engulfment efficiency among all tested proteins. Note that A9 and B1 represent the same graph.

Figure S6.

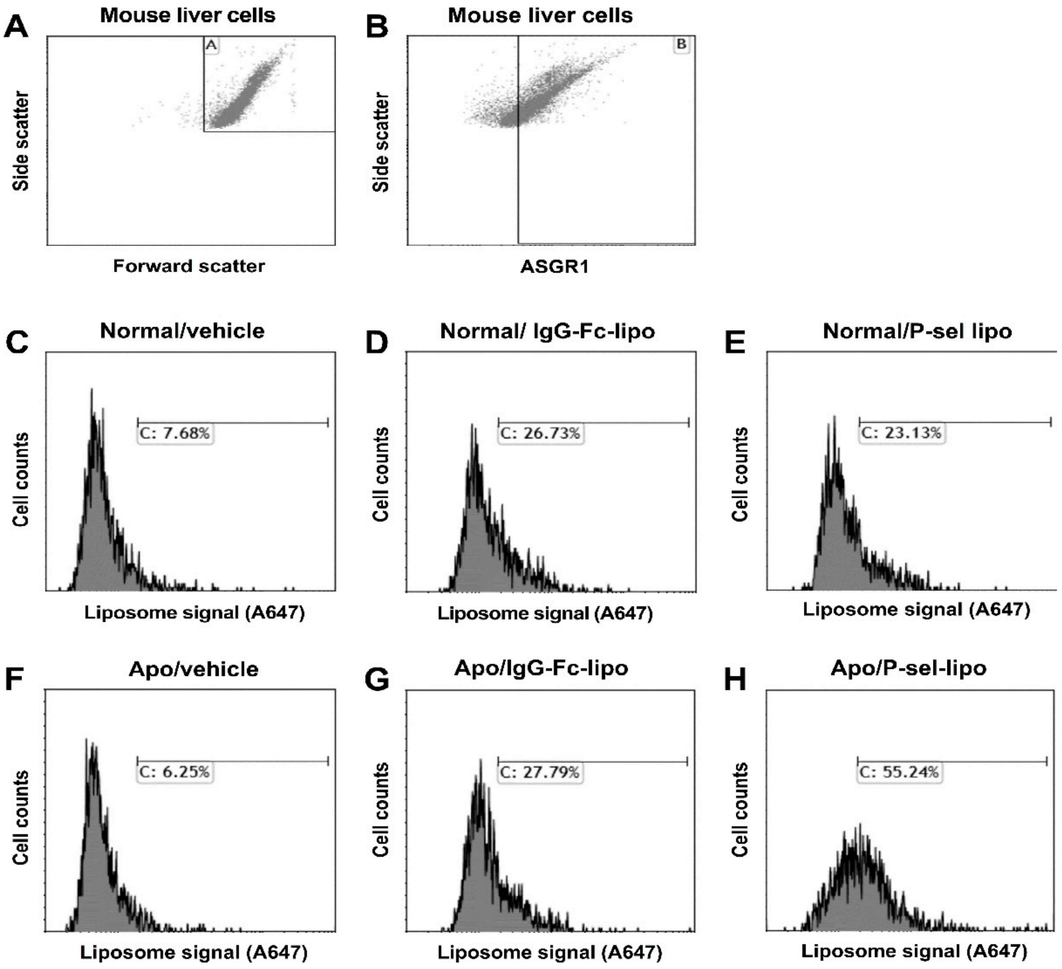

Figure S6. An example illustrates how flow cytometry was used to determine the engagement levels of mouse ASGR1$^+$ hepatocytes with Alexa Fluor 647-loaded liposomes. (A) The liver cell suspension was first gated using forward scatter and side scatter to identify liver cells, which were distinguishable from liposomes due to their larger size. (B) Mouse liver cells were further analyzed for ASGR1 expression, and only the ASGR1$^+$ population was included in subsequent analyses. Cells were treated without (C, D, E, Normal groups) or with (F, G, H, Apo groups) the apoptosis inducer staurosporine, and the engagement of ASGR1$^+$ hepatocytes with fluorescent dye Alexa Fluor 647 (A647)-loaded liposomes was assessed. Flow cytometry was used to detect A647 signals after ASGR1$^+$ hepatocytes were exposed to vehicle (C, F), IgG-Fc-conjugated A647-loaded liposomes (D, G), and P-selectin-conjugated A647-loaded liposomes (E, H).

Figure S7.

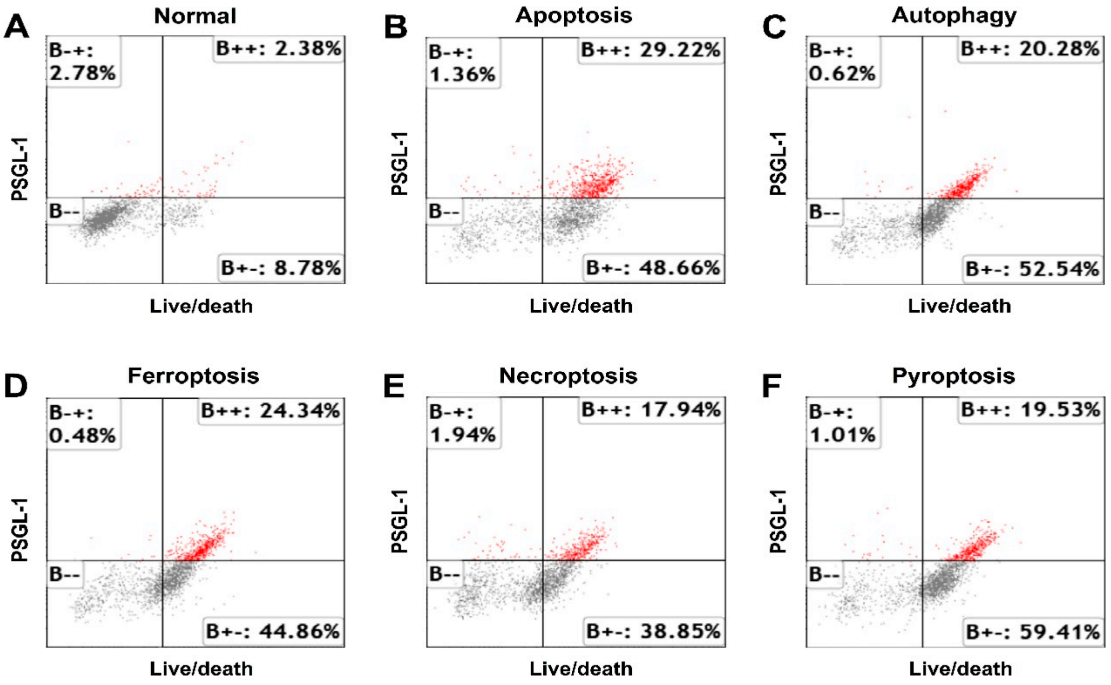

Figure S7. An example of flow cytometry gating used to assess relative surface PSGL-1 expression in Huh-7 cells following treatment with various cell death inducers. The human hepatoma cell line Huh-7 was exposed to vehicle (A), apoptosis inducer (staurosporine) (B), autophagy inducer (rapamycin) (C), ferroptosis inducer (erastin) (D), necroptosis inducer (TNF-α) (E), and pyroptosis inducer (nigericin) (F) for 1 hour. Cells were then double-stained with the Zombie NIR™ Fixable Viability Kit and a fluorescent-labeled anti-PSGL-1 antibody. The PSGL-1 expression in the dying cell population was determined by identifying PSGL-1$^+$ death signal$^+$ double positive population in the upper-right quadrant of the flow cytometry panel.

Figure S8.

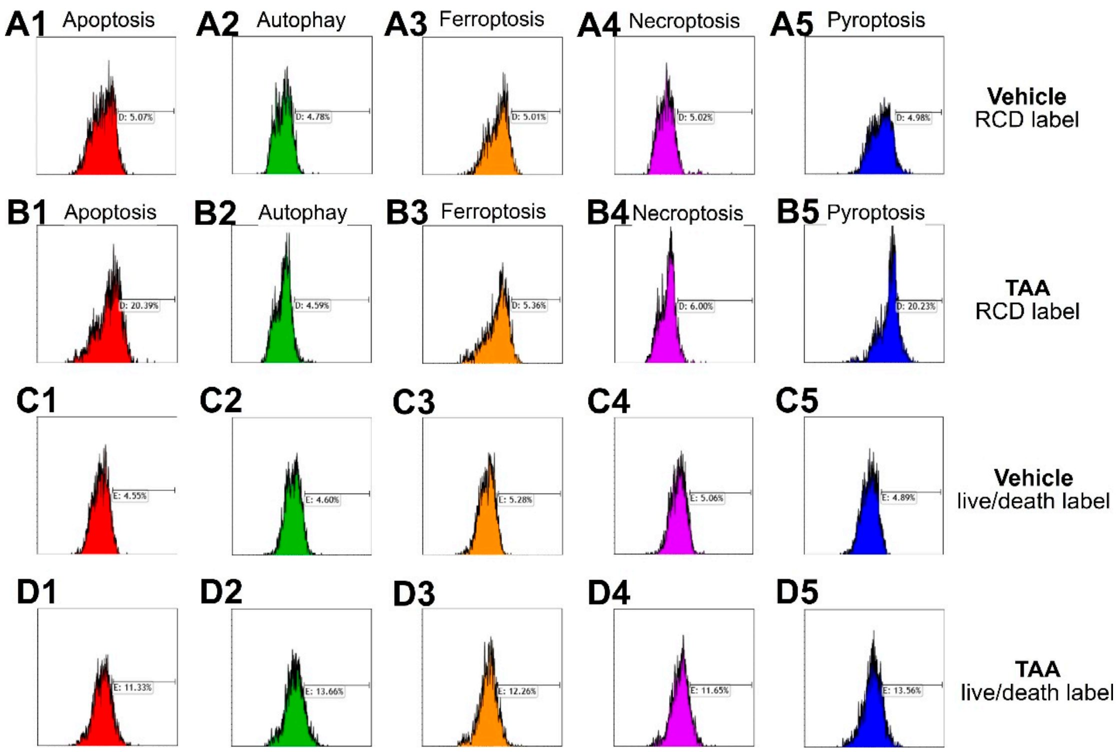

Figure S8. Calculation of the relative increase in regulated cell death (RCD) percentages for mouse liver hepatocytes following thioacetamide (TAA) treatment was performed using flow cytometry. Due to overlapping detection wavelengths, it is not feasible to detect all five RCD pathways (apoptosis, autophagy, ferroptosis, necroptosis, pyroptosis) simultaneously in a single staining sample. Thus, we conducted double staining for each RCD marker along with a live/dead cell status stain as an internal control. For example, hepatocytes isolated from vehicle-treated (A, C) or TAA-treated (B, D) mice were stained for specific RCD markers (A, B) and live/dead status (C, D). The increase in cell death signal (e.g., apoptosis signal B1-A1; △apoptosis) was normalized by the increased death-cell population (e.g., B1-A1/D1-C1; △apoptosis/△total death cell) for each RCD. The sum of B1-A1/D1-C1, B2-A2/D2-C2, B3-A3/D3-C3, B4-A4/D4-C4, and B5-A5/D5-C5 was considered 100%. Therefore, the relative apoptosis percentage was calculated as [B5-A5/D5-C5] / [(B1-A1/D1-C1) + (B2-A2/D2-C2) + (B3-A3/D3-C3) + (B4-A4/D4-C4) + (B5-A5/D5-C5)] × 100%, which was approximately 48.9%. The pie chart results shown in Figure 5A were generated using this formula, based on averaged data from triplicate samples per group.
